# Social defeat stress induces genome-wide 5mC and 5hmC alterations in the mouse brain

**DOI:** 10.1093/g3journal/jkad114

**Published:** 2023-05-25

**Authors:** Janise N Kuehner, Nevin R Walia, Rachel Seong, Yangping Li, Paula Martinez-Feduchi, Bing Yao

**Affiliations:** Department of Human Genetics, Emory University School of Medicine, 615 Michael Street, Atlanta, GA 30322, USA; Department of Human Genetics, Emory University School of Medicine, 615 Michael Street, Atlanta, GA 30322, USA; Department of Human Genetics, Emory University School of Medicine, 615 Michael Street, Atlanta, GA 30322, USA; Department of Human Genetics, Emory University School of Medicine, 615 Michael Street, Atlanta, GA 30322, USA; Department of Human Genetics, Emory University School of Medicine, 615 Michael Street, Atlanta, GA 30322, USA; Department of Human Genetics, Emory University School of Medicine, 615 Michael Street, Atlanta, GA 30322, USA

**Keywords:** 5-methylcytosine (5mC), 5-hydroxymethylcytosine (5hmC), social defeat stress response

## Abstract

Stress is adverse experience that require constant adaptation to reduce the emotional and physiological burden, or “allostatic load”, of an individual. Despite their everyday occurrence, a subpopulation of individuals is more susceptible to stressors, while others remain resilient with unknown molecular signatures. In this study, we investigated the contribution of the DNA modifications, 5-methylcytosine (5mC) and 5-hydroxymethylcytosine (5hmC), underlying the individual differences in stress susceptibility and resilience. Genome-wide 5mC and 5hmC profiles from 3- and 6-month adult male mice that underwent various durations of social defeat were generated. In 3-month animals, 5mC and 5hmC work in parallel and do not distinguish between stress-susceptible and resilient phenotypes, while in 6-month animals, 5mC and 5hmC show distinct enrichment patterns. Acute stress responses may epigenetically “prime” the animals to either increase or decrease their predisposition to depression susceptibility. In support of this, re-exposure studies reveal that the enduring effects of social defeat affect differential biological processes between susceptible and resilient animals. Finally, the stress-induced 5mC and 5hmC fluctuations across the acute-chronic-longitudinal time course demonstrate that the negative outcomes of chronic stress do not discriminate between susceptible and resilient animals. However, resilience is more associated with neuroprotective processes while susceptibility is linked to neurodegenerative processes. Furthermore, 5mC appears to be responsible for acute stress response, whereas 5hmC may function as a persistent and stable modification in response to stress. Our study broadens the scope of previous research offering a comprehensive analysis of the role of DNA modifications in stress-induced depression.

## Introduction

Stress can be described as adverse experiences that contest the emotional and psychological well-being of an individual. Despite stressful events being a part of everyday life, some individuals are more susceptible to these stressors, while others remain resilient. The molecular mechanisms underlying stress susceptibility and resilience remain elusive. In addition, the duration to which the stressor is experienced can also impact susceptibility. Stress can be categorized into different classes according to duration: instantaneous, acute (short-term), chronic (persistent), and longitudinal (re-exposure). The more prolonged the exposure to the stressor, the more likely an individual is to become vulnerable to anxiety and depressive behaviors predisposing them to major depressive disorder (MDD) (Tafet and Bernardini 2003).

Stress-induced depression is robustly affiliated with the hyperactivation of the hypothalamus-pituitary-adrenal (HPA) axis, causing an increase in blood corticosterone levels and neurological phenotypes (Burke *et al*. 2005; Peña-Bautista *et al*. 2020). However, stress-induced alterations to the epigenetic landscape, particularly chemical modifications on the DNA paired with gene expression, are less well characterized. Several pioneering studies investigated how prenatal or early life traumas could epigenetically “prime” an individual to inherently be more predisposed to neuropsychiatric disorders later in life (Meaney and Szyf 2005; Aguilera *et al*. 2010; Tobi *et al*. 2014). Nevertheless, how stress-induced epigenetic alterations in the brain could regulate differences in stress response remains an open question.

Epigenetics broadly refers to heritable changes in gene expression that occur without altering the DNA sequence, through mechanisms such as DNA covalent modifications (Goldberg *et al*. 2007). Importantly, these epigenetic variations can arise as a consequence of environmental factors and have a lasting physiological and pathological impact (Aguilera *et al*. 2010). Previous studies have primarily focused on locus-specific and global histone modification profiles concerning stress. For example, exposure to chronic stress was shown to induce repressive histone methylation at the promoter of specific brain-derived neurotrophic factor (*Bdnf*) splice variants resulting in decreased expression (Tsankova *et al*. 2006). We and others have previously demonstrated the importance of the DNA modifications 5-methylcytosine (5mC) and 5-hydroxymethylcytosine (5hmC) in neurodevelopment and neurodegenerative disorders (Yao and Jin 2014; Kuehner *et al*. 2021; Kaur *et al*. 2022). In terms of DNA modifications in stress response, there is a large body of work concentrating on locus-specific alterations associated with chronic stress. For instance, loss of promoter methylation at the *Crf* gene (Sterrenburg *et al*. 2011), increased expression of the active DNA demethylase inducer *Gadd45b* (Labonte *et al*. 2019) and the persistent upregulation of *Dnmt3a* (LaPlant *et al*. 2010) have all been observed to coincide with stress susceptibility. Ubiquitous knockout of either *Tet1* or *Tet2* DNA demethylase in mice resulted in increased baseline resilience or susceptibility to chronic restraint stress, respectively (Cheng *et al*. 2018). This provides a clear link between epigenetic regulation and differences in stress response. In addition, there is a multitude of evidence supporting the role of epigenetics in neuropsychiatric diseases (Kuehner *et al*. 2019); however, a systematic assessment of global 5mC and 5hmC in relation to various stress durations is needed.

In this study, we generated genome-wide 5mC, 5hmC, and transcriptome profiles from animals that underwent various durations of social defeat. Stress-induced alteration of both DNA modifications was observed with respect to age and duration of stress. We further defined 5mC and 5hmC fluctuations across the acute-chronic-longitudinal time course. Our study broadens the scope of previous research from a locus-specific perspective to a global perspective, offering a comprehensive analysis of the role of DNA modifications in stress-induced depression.

## Materials and methods

### Animals

C57BL/6J male (Jackson Labs, stock #:000664) mice were used as experimental animals. CD1 male retired breeders (Charles River Laboratories, stock #022) that were greater than 4 months in age were used as aggressor animals and singly housed. Experimental animals were grouped and housed with littermates in cages of 2–5 unless otherwise indicated. All animals were maintained on a 12-hour light/dark cycle (lights on from 7:00 AM to 7:00 PM) and provided ad libitum food and water. Social defeats and behavior tests occurred during the beginning of their dark cycle and animals were randomly distributed into control and stress groups. All procedures were approved by Emory University's IACUC committee guidelines and followed the NIH Guide for the Ethical Treatment of Animals.

### Social defeat stresses

Naïve C57BL/6J male experimental animals approximately 3- or 6-months in age were subjected to different durations of social defeat stress (chronic social defeat (CSDS) and longitudinal social defeat (LSDS) 10 consecutive days and acute social defeat (ASDS) 3 consecutive days) in white light as previously described (Golden *et al*. 2011). Every day, each experimental mouse was placed into the home cage of a novel aggressor for ≤5 min and was readily defeated by the aggressor. Animals were immediately removed from the aggressor’s cage at the first sign of blood or other injury. Aggressor mice were retired CD1 breeders that had previously been selected for aggressive behavior (an attack latency less than 60 s upon 2+ consecutive screening tests). After 5 min of physical interaction, experimental and aggressor mice are separated by a clear perforated cage divider so that sensory contact was maintained for 24 h. Each day, experimental mice were exposed to a new aggressor's home cage. Control animals were housed in pair, one on each side of a clear perforated cage divider, rotated daily and never allowed to physically encounter their cage mate. Each variation of the social defeat paradigm (i.e. CSDS, ASDS, and LSDS) was conducted with 4–5 independent cohorts (*n* = 8–10 controls and *n* = 8–10 stressed animals per cohort).

### Social interaction (SI)

Video recordings were used to score interaction and avoidance behaviors toward an unfamiliar CD1 target animal. The arena was a white open field (40 × 40 cm height; Maze Engineers) maintained in complete darkness supplemented with red lamp lights. 24 h after the final defeat, defeated and control mice were introduced into the open field for two consecutive sessions of 2.5 min each. During the first session (“no target”), the open arena contained an empty wire enclosure (enclosure: 10 × 6.5 × 30 cm high; wire mesh: 10 × 6.5 × 8 cm high; Maze Engineers) placed at one end of the arena in the center of the interaction zone. During the second session (“target”), an unfamiliar CD1 target animal was placed in a new wire enclosure. The time spent in the interaction zone and avoidance zone was scored for “no target” and “target” conditions. The social interaction ratio (SIR) was calculated as the time spent in the interaction zone with the target divided by the time spent in the interaction zone without the target. Susceptible animals were defined as having a SIR < 1 and resilient animals had an SIR ≥1. Animals in the LSDS cohorts underwent 2 rounds of SI, one 24 h after the final defeat and a second round 4 weeks later after an “incubation” period. During the incubation period, LSDS animals were singly housed and injuries were treated daily. All videos were scored manually using a stopwatch; however, because repeated defeats can leave visible injuries, it was not always possible for the experimenter to be blinded during scoring thus two independent scores were used and averaged. To avoid artificially inflating the average, animals whose “no target” score time was <1 s or SIR > 300 were removed from the data set. This resulted in the removal of 1 animal from the analysis.

### Sucrose preference testing

Mice were provided 2 bottles daily, 1 with water and the other with 1% (wt/vol) fresh sucrose solution that was counterbalanced for position. Animals were habituated for 3 days with 24-h access to water and sucrose in the experimental cages. Preference testing occurred for 4 days and the animals were given access to water and sucrose after the social defeat session for 12 h. Bottles were weighed the following morning and the sucrose solution was replaced with a water bottle. Sucrose preference was calculated as the percentage of sucrose solution consumed out of the total fluid consumption: 100×sucrose/(sucrose + water).

### Nest building

Nest quality was assessed daily for 24 h following a social defeat. Quality was based on a scale ranging from 0–3. A score of 0 indicated the nestlet had not been touched, 1 indicated minimal shredding; nestlet shape could still be discerned, 2 indicated medium shredding; nestlet shape nearly undiscernible, and 3 indicated full shredding; no nestlet shape could be seen.

### Food consumption

Before the social defeat, food was weighed daily to determine how much was consumed over a 24-h period. As necessary, food was replenished to ensure animals had ad libitum access to food.

### Corticosterone measurement

Blood was collected approximately 36 h after the final defeat except for the ASDS animals whose blood was collected immediately following a defeat. All animals were anesthetized with isoflurane and blood was collected from the axillary (armpit) vessel and put into EDTA-coated tubes (VWR 101094-004) and chilled on ice. Blood was centrifuged for 20 min at 3,000 rpm at 4°C, plasma was collected and stored at −80°C. CORT was measured using the Enzo Life Sciences kit (VWR, ADI-900-097) following the manufacturer's small volume protocol for blood plasma, including diluting samples 1:40 with a steroid displacement reagent solution. Absorption was read at 405 nm in a Synergy H4 hybrid reader (BioTek). Absolute concentrations (pg/mL) were calculated from a standard curve and converted to ng/mL and adjusted for 1:40 dilution.

### RNA isolation and RTqPCR

Brain tissue was placed in TRIzol and homogenized using a hand-held pestle homogenizer and allowed to incubate for at least 5 min. Chloroform was added to the homogenate in 1:5 ratios, the tubes shaken, and allowed to incubate at room temperature for 15 min. Samples were centrifuged at 12,000*g* for 15 min at 4°C. The top aqueous layer was transferred to a clean tube, and the RNA was precipitated in 3 M NaAc pH 5.2 (10:1 ratio), 4 μL of glycogen (5 mg/mL), 100% isopropanol (1:1 ratio) overnight at −37°C. The next day, samples were centrifuged at 15,000 RPM for 20 min at 4°C. The resulting RNA pellet was washed twice in 75% ethanol and centrifuged at 7,500*g* for 10 min at 4°C. The washed RNA pellet was dissolved in nuclease-free water. RNA was quantified by Nanodrop and the quality was confirmed by a gel. RTqPCR was performed using Thermo Fisher's Super Script III First-Strand Synthesis (18080051) kit and the manufacturer's protocol was followed.

### RNA-sequencing

RNA was isolated from brain tissue as described above. Bulk RNA was sent to Admera Health, LLC for library construction and sequencing on an Illumina HiSeq platform.

### DNA isolation

Brain tissue was harvested either immediately (ASDS), 36 h (CSDS), or 4 weeks (LSDS) following the final social defeat and after were immediately frozen on dry ice and stored at −80°C. Tissue was digested in a lysis buffer (10 mM Tris pH 8.0, 5 mM EDTA, 200 mM NaCl, 0.2% SDS) with 30 μL proteinase K (20 mg/mL) and incubated at 55°C overnight. After the overnight digestion, the lysates were brought to room temperature and incubated with 5 μL of RNase A solution (20 mg/mL) for at least 2 hours at room temperature. DNA was extracted by adding an equal volume of buffered phenol:chloroform:isoamyl alcohol (25:24:1 ratio) and centrifuged at 14,000 RPM at room temperature. The supernatant was transferred to clean tubes and 5 μL of 5 M NaCl, 2 μL glycogen, and equal volume of 100% ethanol were added. After overnight incubation at −20°C, DNA was centrifuged at 10,000*g* for 10 min at room temperature and then washed in 70% ethanol. After all, ethanol was removed, the DNA pellet was eluted in nuclease-free water and incubated overnight at 4°C before storing at −20°C before being quantified by Nanodrop.

### 5hmC capture

5hmC capture was performed according to the method described by Kuehner *et al*. (2021). In brief, 5 μg of genomic DNA was sonicated to 300–400 base pairs and 5hmC containing fragments were glucosylated (T4 phage ß-glucosyltransferase enzyme and UDP-6-N3-glucose). The glucosylated fragments were purified and then biotinylated (disulfide biotin linker) and pulled down with Dynabeads MyOne Streptavidin C1 beads. The 5hmC fragments were released from the beads using dithiothreitol and purified for a final time. DNA fragments were eluted in nuclease-free water and quantified by Qubit.

### Methylated DNA immunoprecipitation (MeDIP) and library construction

5mC capture was performed according to the method described by Weber *et al*. (2005) with slight modifications. 3 μg of genomic DNA was sonicated to 300–400 base pairs using a Covaris-focused ultrasonicator. The DNA fragments were then subjected to end repair, A-tailing, adaptor ligation, and USER digestion using the NEBNext Ultra II DNA Library Prep kit for Illumina (New England BioLabs, E7645S) according to the manufacturer's protocol. Following USER digestion and purification, DNA was denatured for 10 min at 95°C and immunoprecipitated overnight at 4°C with 4 μL of either 5mC antibody (Active Motif, 39649) or IgG antibody (Sigma 12–371) in IP buffer (500 mM Tris–HCl, pH 7.4, 750 mM NaCl and 0.25% TritonX). The mixture was then incubated with Protein G-coated Dynabeads for at least 2 h at 4°C, washed with ice-cold IP buffer, and finally washed in ice-cold high salt (300 mM NaCl) IP buffer. After the final washing, the beads were treated with 200 μL digest buffer (1 × TE Buffer, pH 7.4, 0.25% SDS, 0.25% Proteinase K (2.5 mg/mL)), and shaken at 1,000 rpm for 2 h at 55°C. The methylated DNA was recovered by phenol:chloform:isoamyl alcohol (25:24:1) extraction followed by precipitation in 3 × volume of 100% ethanol supplemented with 3 μL glycogen(5 mg/mL) and 15 μL NaAC pH 5.2 overnight at −20°C. The next day, the DNA was pelleted and washed with 75% ethanol and dissolved in nuclease-free water. Illumina indexes were added, PCR enriched, and purified using the NEBNext Ultra II DNA Library Prep kit for Illumina following the manufacturer's protocol.

### Library preparation and high-throughput sequencing

Library preparation and sequencing were performed according to the method described in (Kuehner *et al*. 2021). The NEBNext Ultra II DNA Library Prep kit for Illumina (New England BioLabs, E7645S) was used per the manufacturer's protocol for enriched and unenriched genomic DNA. An Agilent 2100 Bioanalyzer was used to confirm the purity and fragmentation size of the final libraries. Libraries were sequenced pair-end (150 bp) on an Illumina HiSeq platform by Admera Health, LLC. The accession number for the 5mC-seq, 5hmC-seq, and RNA-seq data generated in this paper is GSE223301.

### 5mC/5hmC-seq and RNA-seq data processing

Data mapping and processing were performed as previously described (Kuehner *et al*. 2021). Briefly, bowtie2 (v2.3.5.1) (Langmead *et al*. 2009) was used to map pair-end reads to the mm9 reference genome followed by MACS2 peak calling by default setting to use 1e-5 as the *P*-value cutoff (v 2.1.2) (Zhang *et al*. 2008). Technical replicates were combined using the bedtools (v2.28.0) “window” function with a 300 bp extension applied up and downstream of the peak. Only the common peaks in all the replicates were considered for further analysis. Peaks were then annotated to their corresponding genes using HOMER (Hypergeometric Optimization of Motif EnRichment) (v4.11.1) (Heinz *et al*. 2010) and the flag “-annStat” was added to annotate these peaks to genomic features. Raw RNA-seq reads were aligned to the mouse mm9 genome using TopHat2 (v2.1.0) (Trapnell *et al*. 2012) and differential gene expression analysis was conducted using Cuffdiff (v2.2.1).

### Identification of differentially methylated and hydroxymethylated regions (DMRs and DhMRs)

Using the bedtools “window” function with the same parameters as described above, gained and lost differential regions were identified by comparing control samples to their appropriate stress condition. Peak regions only found in stress samples compared to control were defined as gained, whereas peak regions found to be absent in stress compared to control were defined as lost.

### Bioinformatics analysis

Using susceptible gained and lost DMRs/DhMRs, we compared susceptible and resilient DNA modification profiles. 5mC or 5hmC reads were counted for all the replicates, normalized by their corresponding mapped read counts, averaged and the log_2_ fold change observed between stress and control samples was determined. Susceptible gained and lost DMRs/DhMRs were grouped by the resilient log_2_ fold change and heatmaps were generated to validate the enrichment patterns. This process was repeated using resilient gained and lost DMRs/DhMRs. Susceptible and resilient gained and lost DMRs/DhMRs were annotated to their nearest gene by HOMER and those regions located in intergenic regions were removed from further analysis. Only those genes showing a positive correlation with gene expression data were used for either Gene Ontology (GO) analysis with a false discovery rate <0.05 (Ashburner *et al*. 2000; The Gene Ontology 2019). Genes that gained 5mC or 5hmC and have a positive log_2_ fold change in gene expression will be referred to as concomitantly increasing genes. Whereas genes that lost 5mC or 5hmC and have a negative log_2_ fold change in gene expression will be referred to as concomitantly decreasing genes. To visualize example DMRs/DhMRs, the Integrative Genomics Viewer (IGV) (v2.14.0) was used (Robinson *et al*. 2011).

Comparisons between 3- and 6-month susceptible and resilient animals were made using the corresponding gained and lost DMRs/DhMRS. After taking the averaged normalized read count, the log_2_ fold change observed between stress and control samples was used to categorize the regions into either a group that gained or lost 5mC/5hmC in both 3- and 6-month animals. After annotating the regions to genes, duplicate genes between susceptible gain and loss or resilient gain and loss were removed. Only those genes that showed a positive correlation in 3- and 6-month gene expression (as described above) were used for string analysis (https://string-db.org/) (Szklarczyk *et al*. 2021). The final gene list was then overlapped with the published MDD gene expression data set (Gammie 2021). To identify top potential susceptible and resilient candidate genes, only those genes whose log_2_ fold change in gene expression was greater than 0.25 in both 3- and 6-month animals were considered. Between stress comparisons (i.e. CSDS vs ASDS or CSDS vs LSDS) were done using the bedtools “window” function with the same parameters as described above. Gain and lost ASDS or LSDS DMRs/DhMRs overlapped with either CSDS gain/loss-specific DMRs/DhMRs, respectively. To identify regions that showed either a continual increase, decrease, or return to baseline of 5mC/5hmC across the ASDS-CSDS-LSDS time course, averaged normalized reads were counted and grouped based on the log_2_ fold change observed between corresponding stress and control samples. HOMER gene annotation, RNA-seq correlation, GO analysis and IGV were performed as previously described.

### GO analysis

Functional annotation analysis was conducted in the GO Consortium classification system (http://geneontology.org) (Ashburner *et al*. 2000; The Gene Ontology 2019). We then clustered GO terms to a representative term and plotted by –log_10_FDR (False Discovery Rate) to show their statistical significance.

## Results

### Chronic social defeat stress induces social avoidance in young and mature adult mice

**Fig. 1.**
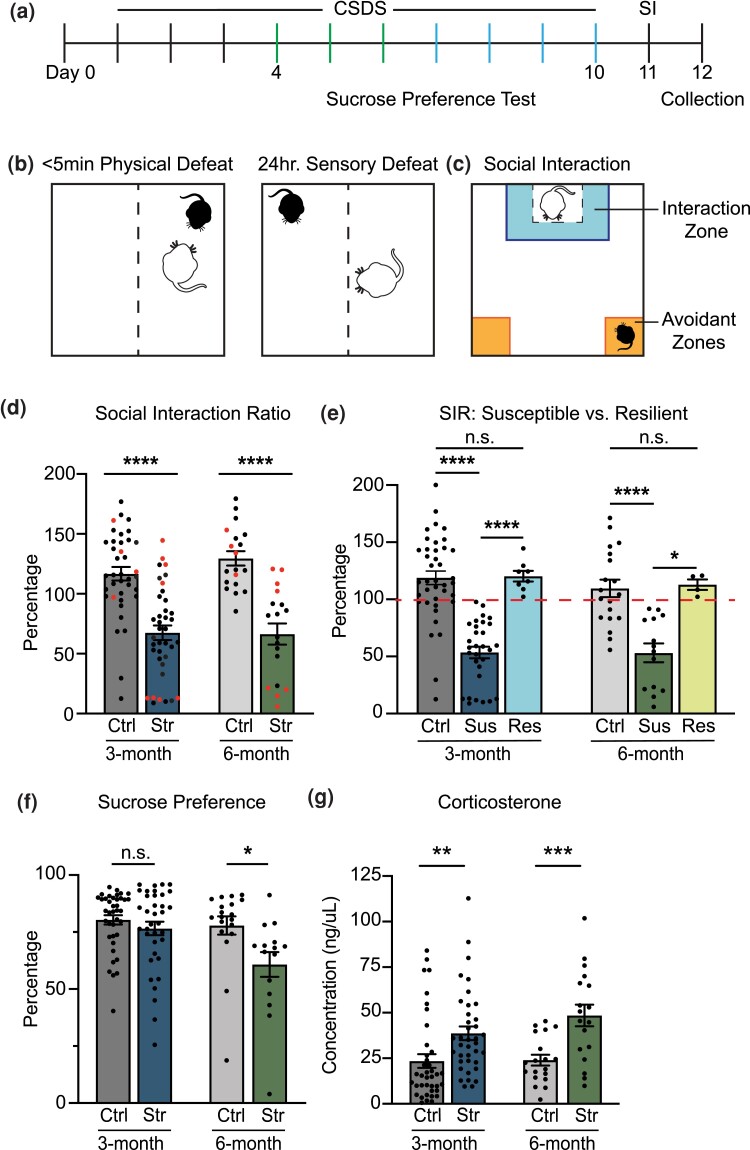
CSDS induces social avoidance in young and mature adult mice. a) Schematic of CSDS paradigm as described in Golden et al. (2011). b) Schematic of social interaction (SI) behavior test. c) Experimental design of CSDS paradigm, behavior tests, and tissue and blood collection. d) Defeated 3- and 6-month-old animals display a significant reduction in social behavior (3-month: n = 39 Ctrl, n = 38 Str, independent cohorts = 4; 6-month: n = 19 Ctrl, n = 18 Str, independent cohorts = 2; Unpaired t-test, ****P < 0.0001 vs *aged matched and littermate controls*). Red dots indicate randomly selected animals used for downstream 5mC and 5hmC enrichment and RNA-Seq experiments. e) Social interaction ratios (SIR). Stress-susceptible (SIR < 100) and stress-resilient (SIR > 100) 3- and 6-month animals as indicated by the dashed red line (*P < 0.05 and ****P < 0.0001 in Tukey's post hoc *test after One-way ANOVA*). f) Sucrose preference testing for anhedonic-like behavior in 3- and 6-month animals (Unpaired t-test, *P < 0.05). g) Blood corticosterone was significantly elevated in both 3- and 6-month animals following CSDS (unpaired t-test, **P < 0.01 and ***P < 0.001). Abbreviations: CSDS: chronic social defeat stress, SI: social interaction, SIR: social interaction ratio, Ctrl: control, Str: stress, Sus: susceptible, Res: resilient.

Chronic social defeat stress (CSDS) is an ethologically relevant paradigm that has been shown to induce depressive and anxiety-like behaviors in rodents (Golden *et al*. 2011). Over the span of 10 days, naïve 3-month (young) and 6-month (mature) mice were repeatedly subjected to bouts of physical defeat by an aggressor (Fig. 1a and b). Defeats were followed by a 24-h sensory stress in the aggressor's home cage through a clear, perforated divider. Nest building and food consumption were measured daily as indicators of distress in the animals (Supplementary Fig. 1a–d). Interestingly, during the first 2 days of defeat, more low-quality nests and a reduction in food consumption were observed in both age groups. After the second defeat, nest quality improved and significantly more food was consumed for the remainder of the experiment. These behaviors uncovered an initial acute response followed by stress adaptation, recapitulating stress habituation behaviors, or allostasis, observed in humans (McEwen 1998). Social avoidant-like behavior was measured using the social interaction (SI) test (Fig. 1c). Three- and 6-month stressed animals exhibited social avoidant behavior compared to controls (Fig. 1d), as indicated by the significant decrease in time spent interacting with the target and the strong preference toward the avoidant zones (Supplementary Fig. 1e and f). Out of all the stressed animals, just over 20%, regardless of age, displayed social behavior indistinguishable from control animals and were designated as resilient (Fig. 1e). Sucrose preference testing suggested that age may play a role in the development of anhedonic-like behavior. While sucrose consumption did not significantly change in the 3-month animals, we did observe increased anhedonic-like behavior in the 6-month animals (Fig. 1f). Finally, blood corticosterone levels were measured approximately 36 h after the final defeat and a significant increase in corticosterone was observed in defeated animals (Fig. 1g). Cumulatively, our data confirm that the CSDS paradigm induces physiological and biological symptoms of depression as well as distinguishes between stress-susceptible and resilient animals, which can be used to elucidate how epigenetics contribute to stress response.

### Global characterization of susceptible and resilient DMRs and DhMRs in young and mature adult mice

**Fig. 2.**
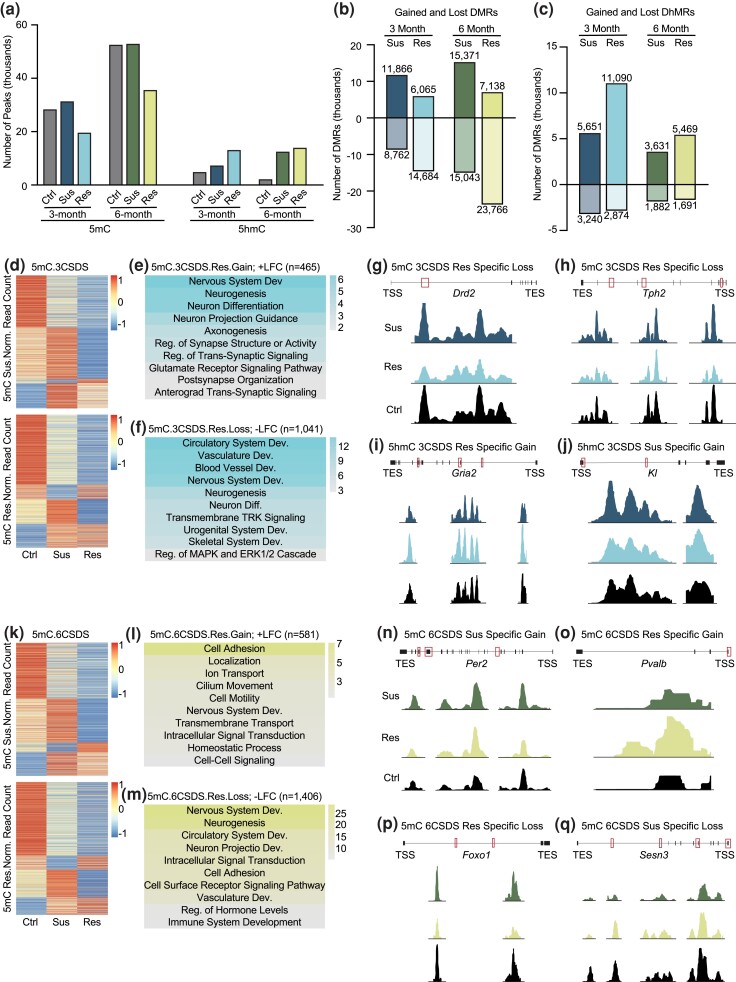
Characterization of susceptible and resilient chronic stress-induced DMRs and DhMRs. a) Number of 5mC and 5hmC peaks identified in control (Ctrl), susceptible (Sus), and resilient (Res) 3- and 6-month animals. b and c) Number of gain and lost 5mC (differentially methylated regions, DMRs) (b) and 5hmC (differential hydroxymethylated regions, DhMRs) (c) peaks in stress-susceptible and resilient 3- and 6-month animals. d and k) Heatmap of susceptible and resilient DMRs from 3-month (d) and 6-month (k) chronically stressed animals, where the color scale represents normalized 5mC read counts. e and f) Representative GO analysis of the genes from 3-month (e and f) and 6-month (l and m) stress-resilient animals that either concomitantly increased in 5mC and gene expression (e and l. Gain; +LFC) or concomitantly decreased in 5mC and gene expression (f and m. loss; –LFC). Color scale represents -log_10_ FDR. GO analysis for remaining 5mC and 5hmC analysis are in Tables 1 and 2. g–j and n–q) Normalized 5mC or 5hmC counts at specific peak regions identified in 3-month (g–j) CSDS animals: *Drd*2 (g), *Tph2* (h), *Gria2* (i) and *Kl* j) and 6-month (n–q.) CSDS animals: *Per2* (n), *Pvalb* (o), *Foxo1* (p) and *Sesn3* (q). Abbreviations: DMRs: differentially methylated regions, DhMRs: differential hydroxymethylated regions, Norm.: normalized, LFC: log fold change, Dev.: development, Reg.: regulation, TRK: tyrosine receptor kinase, TSS: transcription start site, TES: transcription end site.

Given those stress-susceptible and resilient animals were obtained, we investigated how DNA modifications, including 5mC and 5hmC, could contribute to differences in stress response and be responsible for the increased stress sensitivity observed with age. Using established 5mC and 5hmC enrichment methods (5mC-seq and 5hmC-seq, respectively) (Kuehner *et al*. 2021), we generated genome-wide 5mC and 5hmC profiles of the cortex, an important stress-responding brain region (Arnsten 2009). Concerning 5mC, we found that 6-month animals overall had more 5mC peaks compared to 3-month animals (Fig. 2a). Notably, stress-resilient animals regardless of age, have approximately 1.5 times less 5mC and the highest level of 5hmC compared to control and susceptible animals. This suggests a trend toward global hypo-methylation and hyper-hydroxymethylation that is strongly associated with resilient animals. Enrichment analysis revealed expected genomic patterning for both 5mC and 5hmC peaks (Szulwach *et al*. 2011; Taiwo *et al*. 2012) (Supplementary Fig. 2a–d). 5mC is primarily enriched in exons while 5hmC is distributed across gene bodies with strong depletion in intergenic regions. Overall, stress did not induce any global changes to the enrichment patterns of either 5mC or 5hmC compared to expected values, suggesting that after the initial defeats, the epigenetic landscapes could have stabilized as a stress adaptation, similar to the feeding and nesting behaviors.

**Table 1.** Biological processes corresponding to the gained/lost DMRs from 3- and 6-month susceptible and resilient animals.

Animal (numb genes gain/loss)	5mC gain; +LFC (−log 10 FDR)	5mC loss; –LFC (−log 10 FDR)
3-month susceptible (*n* = 947/281)	Nervous System Dev. (10.2) Neurogenesis (9.8) Generation of Neurons (7.8) Neuron Diff. (6.5) Cell Adhesion (6.1) Neuron Projection Dev. (3.2) Reg. of Trans-Synaptic Signaling (2.9) Synapse Org. (2.2) Cell Surface Receptor Signaling Pathway (2.1) Reg. of Neurotransmitter Levels (2.0)	Cell Adhesion (9.4) Cell Diff. (6.0) Cell Junction Org. (4.2) Nervous System Dev. (3.9) Neurogenesis (3.0) Circulatory System Dev. (2.9) Blood Vessel Morph. (2.8) Vasculature Dev. (2.8) Neuron Projection Morph. (1.7) Cell Surface Receptor Signaling Pathway (1.4)
3-month resilient (*n* = 465/1,041)	Nervous System Dev. (6.4) Neurogenesis (5.7) Neuron Diff. (5.6) Neuron Projection Guidance (4.1) Axonogenesis (3.6) Reg. of Synapse Structure or Activity (3.3) Reg. of Trans-Synaptic Signaling (3.1) Glutamate Receptor Signaling Pathway (2.1) Post-synapse Org. (1.9) Anterograde Trans-Synaptic Signaling (1.8)	Circulatory System Dev. (14.1) Vasculature Dev. (12.9) Blood Vessel Dev. (12.8) Nervous System Dev. (11.9) Neurogenesis (8.6) Neuron Diff. (6.9) Transmembrane TRK Signaling (6.9) Urogenital System Dev. (6.7) Skeletal System Dev. (5.7) Reg. of MAPK and ERK1/2 Cascade (2.4)
6-month susceptible (*n* = 1,014/697)	Transmembrane Transport (6.5) Small Molecule Metabolic Process (3.7) Response to Wounding (2.9) Homeostatic Process (2.8) Ion Transmembrane Transport (2.5) Leukocyte Activation (2.2) Reg. of Response to Stress (1.8) Activation Involved in Immune Response (1.8) Inflammatory Response (1.7) Reg. of Cytokine Production (1.5)	Nervous System Dev. (21.0) Neurogenesis (19.9) Neuron Diff. (17.4) Generation of Neurons (16.9) Neuron Projection Morph. (14.4) Circulatory System Dev. (13.4) Axon Guidance (12.4) Reg. of Cell Communication (12.1) Axonogenesis (11.9) Muscle Structure Dev. (9.3)
6-month resilient (*n* = 581/1,406)	Cell Adhesion (7.2) Localization (3.5) Ion Transport (3.2) Cilium Motility (2.6) Cell Motility (2.6) Nervous System Dev. (2.4) Transmembrane Transport (2.2) Intracellular Signal Transduction (2.0) Homeostatic Process (1.9) Cell–Cell Signaling (1.3)	Nervous System Dev. (29.3) Neurogenesis (26.5) Circulatory System Dev. (18.2) Neuron Projection Dev. (17.7) Intracellular Signal Transduction (16.5) Cell Adhesion (15.5) Cell Surface Receptor Signaling Pathway (14.9) Vasculature Dev. (12.0) Reg. Hormone Levels (6.2) Immune System Dev. (5.3)

GO analysis on the 5mC regions that either concomitantly increase (gain; +LFC) or decrease (loss; –LFC) in 5mC and gene expression (*n* = number of concomitantly increase/decrease genes). The table is a continuation of Fig. 2.

**Table 2.** Biological processes corresponding to the gained/lost DhMRs from 3- and 6-month susceptible and resilient animals.

Animal (numb genes gain/loss)	5hmC gain; +LFC (−log 10 FDR)	5hmC loss; –LFC (−log 10 FDR)
3-month susceptible (*n* = 712/343)	Nervous System Dev. (11.3) Reg. of Signal Transduction (9.5) Neuron Diff. (8.8) Neuron Projection Dev. (7.5) Cytoskeleton Org. (5.7) Macromolecule Mod. (4.3) Wnt Signaling Pathway (3.7) Homeostatic Process (3.5) Cell Surface Receptor Signaling (2.9) Neuromuscular Process (2.0)	Developmental Process (8.3) Cell Differentiation (5.6) Nervous System Dev. (5.5) Reg. of Signal Transduction (4.8) Cell Junction Org. (3.6) Cytoskeleton Org. (2.9) Phosphorylation (2.8) +Reg. of Cell Communication (2.4) Protein Modification Process (2.2) Macromolecule Localization (2.2)
3-month resilient (*n* = 1,719/228)	Nervous System Dev. (23.6) Macromolecule Mod. (21.9) Neuron Projection Dev. (19.1) Neuron Diff. (16.7) Chemical Synaptic Transmission (13.4) Synapse Org. (12.3) Metabolic Process (10.8) Reg. of Dendrite Dev. (7.1) Synaptic Signaling (5.2) Vesicle Mediated Transport in Synapse (5.1)	Reg. of Cell Migration (6.5) Transmembrane Receptor TK Signaling (5.9) Cell Surface Receptor Signaling (5.4) Angiogenesis (5.0) Blood Vessel Morph. (4.1) Vasculature Dev. (3.8) Reg. of Cell Junction Assembly (3.8) Reg. of Cell Communication (3.7) Reg. of Catalytic Activity (3.0) Reg. of MAPK cascade (3.0)
6-month susceptible (*n* = 313/213)	Reg. of Cell Communication (2.1) Reg. of Signaling (1.9) Reg. of Signal Transduction (1.7) Developmental Process (1.6) Reg. of Insulin Secretion Involved in Response to Glucose Stimulus (1.4) Wnt Signaling Pathway (1.4) Homeostatic Process (1.4) Reg. of Response to Stimulus (1.4) Cell–Cell Signaling by Wnt (1.3)	Intracellular Signal Transduction (6.8) Reg. of Cell Diff. (5.7) Nervous System Dev. (4.5) Protein Phosphorylation (4.4) Reg. of Macromolecule Mod. (4.2) Reg. of Catalytic Activity (4.1) +Reg. of Hydrolase Activity (3.9) Reg. of Cell Adhesion (3.5) Reg. of GTPase Activity (3.3) Reg. of Gene Expression (2.1)
6-month resilient (*n* = 610/116)	Metabolic Process (7.8) Reg. of Cell Diff. (4.2) Protein Localization (4.2) Reg. of Cell Communication (3.8) Protein Modification Process (3.6) Catabolic Process (2.8) Nervous System Dev. (2.8) Cellular Homeostasis (2.3) Reg. of Cell Death (1.9) Energy Reserve Metabolic Process (1.6)	Reg. of Cellular Process (3.2) Reg. of Biological Process (3.1) Reg. of Metabolic Process (2.7) Reg. of Macromolecules (2.1) Reg. of Signaling (1.8) Reg. of Cell Communication (1.8) Dephosphorylation (1.5) Golgi Plasma Membrane Protein Transport (1.5) Vesicle Cytoskeletal Trafficking (1.5) Reg. of Dev. Process (1.3)

GO analysis on the 5hmC regions that either concomitantly increase (gain; +LFC) or decrease (loss; –LFC) in 5hmC and gene expression (*n* = number of concomitantly increase/decrease genes). The table is a continuation of Fig. 2.

We next identified differentially methylated and hydroxymethylated regions, DMRs and DhMRs respectively, in 3- and 6-month stress-susceptible and resilient animals by comparing each stress condition to its corresponding control using our published computational approach (Kuehner *et al*. 2021). Regardless of age, stress-susceptible animals consistently gained more and lost fewer DMRs compared to resilient animals (Fig. 2b). Furthermore, the opposite was observed for DhMRs, where the resilient animals gained more DhMRs (Fig. 2c). To determine if the cyclic conversion of 5mC to 5hmC to unmodified C could be important for promoting the resilient phenotype, we compared gained and lost DMRs to lost and gained DhMRs, respectively (Supplementary Fig. 2e and f), but observed very little overlap. In the brain, it has previously been demonstrated that fluctuations of 5hmC occur independent of 5mC level changes (Hahn *et al*. 2013). This data supports 5hmC as a de novo and independent epigenetic mark in the mammalian brain, so we separately analyzed 5mC and 5hmC in the following sections. Collectively, our data support that chronic stress induces alterations to the 5mC and 5hmC landscape of the cortex and that these changes correspond to differences in stress response.

Although, 5mC is generally associated with gene repression, particularly at promoter regions, in the mammalian genome, both 5mC and 5hmC within the gene body have been reported to positively correlate with gene expression (Suzuki and Bird 2008; Ball *et al*. 2009). It has also been well-established that intragenic 5hmC is positively correlated with gene expression (Yao and Jin 2014; Kuehner *et al*. 2021; Kaur *et al*. 2022). Thus, we focused on the positive association between both types of DNA modifications in the gene bodies and gene expression throughout our entire study to mechanistically link the contribution of DNA modifications in gene expression. The percentage of concomitant genes (5mC or 5hmC positively correlated with gene expression) was indicated (Supplementary Fig. 2g). In general, we found roughly 40% of concomitant upregulated genes with a gain of DNA modifications, and 58% of concomitant downregulated genes with loss of DNA modifications among the total expressing genes in the mouse brain. An example scatterplot showed the correlation between 5hmC and gene expression changes in 3-month stress-susceptible mice using a loss of DhMR regions. The log fold change (LFC) values (stress vs control) of 5hmC read count and their annotated gene expression were indicated, and concomitant genes were highlighted (Supplementary Fig. 2h, blue dots indicating concomitant genes). The ratio between concomitant and nonconcomitant genes from Supplementary Fig. 2h was also calculated (Supplementary Fig. 2i). To characterize susceptible- and resilient-related DNA modifications that correspond to age, we grouped the regions into either susceptible or resilient 5mC gain or 5mC loss groups (Fig. 2d and k) and correlated these changes in DNA modifications to concomitant changes in gene expression. Using GO analysis, we found that 3-month concomitantly increasing DMRs and DhMRs from susceptible and resilient animals show a strong bias toward the nervous system process (Fig. 2e and Tables 1 and 2). For both susceptible and resilient gained DMRs and resilient gain DhMRs, the nervous system bias appears to focus specifically on synaptic function. Meanwhile, the susceptible gain DhMRs also function in signaling pathways. Three-month concomitantly decreasing DMRs and DhMRs reside in genes involved in cellular signaling, communication, and vasculature morphogenesis, regardless of the stress response (Fig. 2f and Tables 1 and 2). Moreover, an inspection of 3-month resilient associated DMRs and DhMRs, we discovered several genes with critical roles in neurotransmitter activity such as the dopamine receptor gene *Drd2* (Fig. 2g), the serotonin synthesizing enzyme *Tph2* (Fig. 2h) and the glutamate receptor gene *Gria2* (Fig. 2i). Additionally, susceptible-specific regions that gained 5hmC were found in the klotho gene *KI* (Fig. 2j), which has been linked to chronic stress-induced depression through its modulation of the glutamate receptor subunit GluN2B (Wu *et al*. 2022). Overall, these findings suggest that in 3-month animals, intragenic 5mC and 5hmC are working in parallel, functioning in similar biological processes in response to chronic stress, and do not appear to distinguish between stress-susceptible and resilient phenotypes. The corresponding IGV view of those regions in 6-month animals is shown in Supplementary Fig. 3a–d.

Compared to 3-month animals, 6-month animals displayed more distinctive 5mC and 5hmC enrichment patterns in response to chronic stress. We found that concomitantly increasing DMRs in 6-month susceptible animals are involved in immune response processes such as leukocyte activation, inflammatory response, and the regulation of cytokine production, whereas DMRs from mature resilient animals are associated with cell–cell signaling, cilium, and cell motility (Fig. 2l and Table 1). With regards to DhMRs, 6-month susceptible animals expressed gene enrichment in signaling pathways or cascades, one of which was the regulation of insulin in response to glucose stimulation (Table 2). Resilient DhMRs are involved in basic cellular processes with an emphasis on metabolic and protein-modifying processes (Table 2). Based on the data presented, the concomitant increase of DNA modifications and gene expression show distinct enrichment patterns between 6-month susceptible and resilient animals. Gain of either 5mC or 5hmC in mature susceptible animals is associated with canonical stress-responding processes (i.e. immune, wounding, insulin and glucose, homeostasis), whereas the gain of 5mC or 5hmC in mature resilient animals is associated with basic cellular signaling (cell–cell signaling and metabolism). For the concomitantly decreasing 6-month DMRs, both susceptible and resilient animals showed a bias for general nervous system processes, but resilient animal DMRs are also involved in signaling, vasculature development, hormone regulation, and the immune system (Fig. 2m and Table 1). Regarding susceptible and resilient DhMRs, both annotated genes primarily function in basic cellular and enzymatic activities (Table 2). Corroborating chronic stress affecting signaling pathways, DMRs were found in the circadian rhythm gene *Per2* (Fig. 2n). We also observed an increase in promoter methylation of the *Pvalb* gene (Fig. 2o), which has previously been observed in patients with MDD (Thaweethee-Sukjai *et al*. 2019). DMRs were also observed in the transcription factor *Foxo1* (Fig. 2p) which is known to directly regulate the expression of the sestrin gene *Sesn3* (Fig. 2q) (Chen *et al*. 2010). Moreover, *Foxo1* and *Sesn3* function in insulin and glucose energy metabolism (Lee *et al*. 2012; Schwartz *et al*. 2013), the main energy sources of the brain. Given that the demand for energy metabolites increases during extended periods of stress to maintain a balanced allostatic state, perhaps resilience in mature animals is associated with improved regulation of energy homeostasis. The corresponding IGV view of those regions in 3-month animals is shown in Supplementary Figure 3e–h.

### Comparison between shared DMRs and DhMRs in young and mature adult mice

**Fig. 3.**
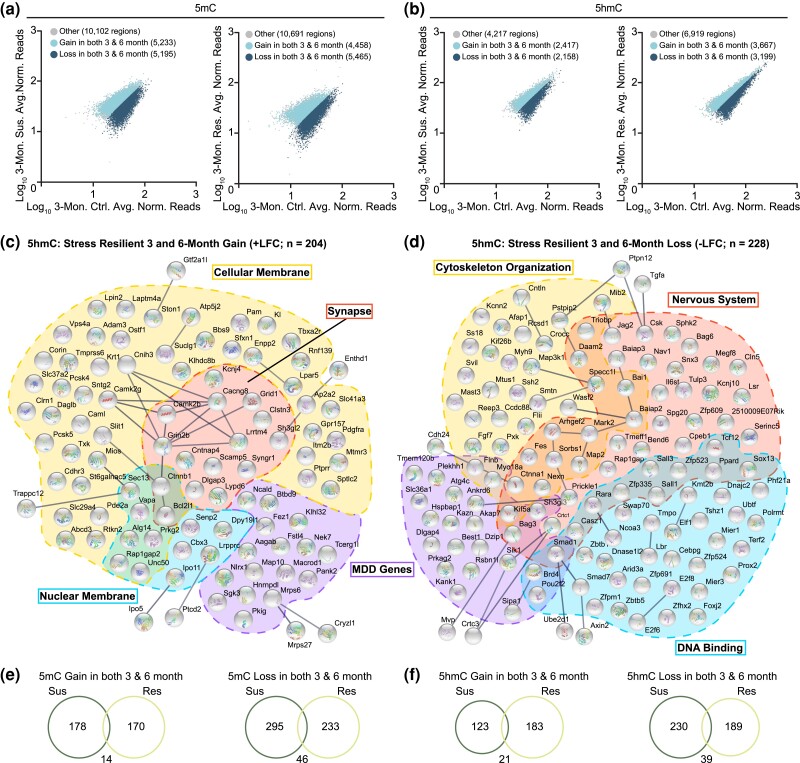
Comparison of shared DMRs and DhMRs in 3- and 6-month animals. a and b) 5mC (a) and 5hmC (b) regions that are gained or lost in 3-month susceptible or resilient animals. Colored dots indicate 6-month regions. c and d) Representative STRING analysis of the shared genes between 3- and 6-month stress-resilient animals that either concomitantly increase (c) or decrease (d) in 5hmC and gene expression. Analysis of the remaining 5mC and 5hmC STRINGs are in Tables 3 and 4. e and f) The overlap of shared 3- and 6-month genes observed between susceptible and resilient animals.

We next sought to directly compare our 3- and 6-month chronically stressed animals and identify shared susceptible or resilient DMRs and DhMRs. Using the gain and loss of susceptible and resilient DMRs and DhMRs identified in 3-month animals (Fig. 2b and c), we determined which of those regions were also gained or lost in susceptible and resilient 6-month animals (Fig. 3a and b), respectively. Given that the 6-month animals displayed increased stress susceptibility, we wanted to determine if 6-month differential regions had a more drastic gain or loss of either DNA modification. Close to 90% overlap between 3- and 6-month DMRs and DhMRs was observed (Supplementary Fig. 3i and j), suggesting that when intragenic 5mC or 5hmC was gained or lost in response to stress in 3-month animals, it was also gained or lost to the same magnitude in 6-month animals. This indicates that with age, there is not a more severe gain or loss of either DNA modification, suggesting that the shared regions are likely not the principal drivers for increased stress susceptibility in mature animals, and are instead contributing to the overall stress response. To investigate the biological functions of the common DMRs and DhMRs, we correlated the corresponding genes to our gene expression data and only considered those genes that had a concomitant increase or decrease in both 3- and 6-month animals and used STRING to cluster the genes into functional groups (Fig. 3c and d and Tables 3 and 4). In addition, we overlapped our genes with a large-scale published MDD gene list (Gammie 2021). Finally, we validated that our susceptible and resilient DNA profiles are independent of each other by overlapping the concomitantly increasing or decreasing susceptible and resilient gene lists and found marginal overlap (Fig. 3e and f).

**Table 3.** Gene list of annotated DMRs shared between 3- and 6-month animals.

	No. of genes in data set	Genes in network	Biological process
Stress-susceptible 3 and 6-month loss –LFC	341	*Agtr1a, Akap13, Asxl2, Bicc1, Bmp7, Cacna1c, Cad, Cdkn1a, Col4a2, Dchs1, E2f8, Ece1, Eln, Epas1, Ephb3, Erbb2, Flt1, Gatad2a, Greb1, Heg1, Heyl, Hif3a, Loxl2, Megf8, Myh9, Myom1, Nebl, Nfatc1, Ngfr, Notch2, Nrp1, Pdlim5, Ppard, Prokr1, Ptch1, Rps6ka2, Sgcd, Smad1, Svep1, Syk, Tenm4, Zfpm1*	Heart/circulatory system
		*Abcc2, Ano1, Atp8a2, Cacna1c, Cacna1d, Cacna1h, Cnnm4, Glp1r, Grid2, Hephl1, Kcnb1, Kcnh7, Klhl3, Nfatc1, Panx2, Piezo1, Plch1, Rasa3, Rimbp2, Slc10a7, Slc16a7, Slc1a5, Slc4a11, Slc6a1, Slc6a11, Slc6a20a, Slc8a2, Slco2a1, Slco2b1, Spx, Steap3, Syk, Xkr7, Trpc2*	Ion transport
		*Abca2, Adam22, Adcy6, Atp8a2, Bcl2l11, Bmp7, Boc, Bsn, Btbd3, Celsr3, Clmn, Dchs1, Dclk1, Egflam, Eif2ak4, Ephb3, Erbb2, Farp1, Fat3, Fos, Foxo3, Foxo6, Gas7, Gatad2a, Gpr98, Grid2, Hap1, Heyl, Ikbkb, Ipmk, Itsn1, Kdr, Lpar3, Mdga1, Megf8, Mertk, Mturn, Myo7a, Nes, Ngfr, Nrp1, Obsl1, Pak4, Pdlim5, Plxnc1, Ppard, Prex1, Ptch1, Ptprs, Rab17, Rap1gap2, Ret, Sema3d, Slc6a11, Smad1, Sox13, Spen, Srrm4, St6gal1, Tenm4, Tmem108, Trim71, Tshr, Ttbk2, Vax1, Zeb1, Zswim4*	Nervous system development
		*Abca2, Agtr1a, Akap13, Arfgap3, Bcl2l11, Bmp7, Ddx11, Dusp18, Elf2, Emp2, Ephb3, Erbb2, Farp1, Flt1, Git2, Gpr98, Gpsm2, Heg1, Hip1, Ikbkb, Itsn1, Kdr, Kiaa1324, Ksr1, Lats2, Lpar3, Nek10, Nes, Ngfr, Nrp1, Pak4, Parp8, Plxnc1, Prex1, R3hdml, Rabep2, Rap1gap2, Ret, Sash1, Sgsm1, St18, Stk10, Tbc1d1, Tbc1d2, Tbc1d4, Timp3, Tshr, Uaca*	Catalytic activity
		*Akap13, Ankrd6, Cacna1d, Cdkn1a, Celsr3, Cep128, Clmn, Clstn2, Dclk1, Dhx8, Dip2a, Elfn2, Emp2, Flt1, Fos, Gpd2, Gpr3, Heg1, Heyl, Hip1, Itpkb, Itsn1, Jmjd1c, Kcnb1, Kcnh7, Kif13b, Klf12, Mturn, Nrp1, Olfm2, Osbpl3, Parp8, Pde1c, Phldb2, Plekhg3, Prima1, Ptch1, Ptprs, Rap1gap2, Rasa3, Rimbp2, Rnd3, Sgcd, Slc16a7, Smad1, Sorbs1, Spen, Srrm4, St18, St6gal1, Tmem151b, Tshr, Uaca, Wdr7*	MDD
Stress-susceptible 3 and 6-month gain +LFC	192	*Acot7, Adam32, Ak8, Aldh1l1, Ank3, Bckdhb, Ccdc91, Cd83, Cdc34, Celf2, Cpn1, Ctss, Dnah1, Dtnbp1, Echdc3, Efhd1, Fabp4, Fsip1, Fyb, Hao1, Hpn, Hydin, Kiz, Lck, Lsp1, Map2k5, Mlycd, Msra, N4bp3, Neu2, Ngf, Osbp2, Pdlim3, Pecr, Pex7, Plb1, Plod2, Prkag2, Rab3ip, Ranbp17, Rarres2, Rbm25, Rbm47, Rbp1, Rbpms, Rgs10, Rrp7a, Siglec1, Slc45a3, Smarca4, Soat2, Socs2, Spata19, Spatc1l, Taf1b, Tagln2, Tdrd9, Terf2, Thyn1, Wipi1, Zbtb20*	Endocrine gland expressing genes
		*Acot7, Bckdhb, Bend5, Ccdc91, Csrp1, Dnah1, Dnajc12, Efhd1, Foxp1, Hs3st4, Inpp5a, Kcnip3, Kiz, Map2k5, Mccc2, Ogfod3, Olfm1, Pdlim3, Pdpn, Prkag2, Rgs10, Scnn1a, Sftpc, Stk39, Taf1b, Vit, Zbtb20*	MDD
Stress-resilient 3 and 6-month loss –LFC	279	*Agtr1a, Alx4, Amotl1, Bbs2, Bmp6, Bmp7, Casq2, Ccm2l, Cdh5, Cntfr, Col4a1, Col4a2, Dab2, Dchs1, Dlx3, Dtnbp1, Eln, Epas1, Ets1, Fgf1, Fli1, Flt1, Frem2, Gpr116, Gpr124, Heg1, Hhip, Hmga2, Kat2b, Kdr, Lmod3, Ltbp1, Megf10, Myh11, Myom1, Ncor2, Nfatc1, Ngfr, Notch1, Pdlim5, Pgm5, Prdm1, Ptprb, Ryr3, Sall1, Stat1, Tead1, Tfap2a, Tmem67, Tnfrsf1a, Tpm2, Trp53bp2*	Heart/circulatory development
		*Bach2, Bmp6, Cd276, Cdh17, Chst3, Crtc3, Csf1, Dab2, Epas1, Ets1, Fli1, Flt1, Fyn, Gpr116, Hck, Ikzf1, Ikzf3, Il31ra, Jarid2, Kdr, Mitf, Mpl, Nbeal2, Nfatc1, Nod1, Orai1, Pdcd1lg2, Plcg2, Prdm1, Rtp4, Stat1, Stat2, Syk, Tirap, Usp18*	Immune response
		*Ankrd6, Cacna1d, Cacng4, Capn2, Ccdc88c, Cdh5, Chst3, Col4a1, Col4a2, Csf1, Csnk1g2, Drd2, Ezr, Fam83d, Fgf1, Flt1, Fyn, Fzd6, Gdnf, Gng12, Gpr124, Hck, Hmga2, Igfbp1, Insrr, Irs1, Itga6, Kank2, Kdr, Lamb2, Lamc1, Map3k1, Ncf4, Ncor2, Nfatc1, Ngfr, Nod1, Notch1, Osmr, Pde8a, Pik3ap1, Plcg2, Ptpn7, Rps6ka1, Sh2d2a, Spry4, Stat1, Syk, Timp3, Tirap, Tnfrsf1a, Tnn, Trp63*	Signaling pathways (PI3k-Akt, MAPK, ERK1/2, Tyrosine kinase, Wnt, steroid hormone)
		*Adarb2, Amotl1, Ankrd6, Bcas1, Cabin1, Cacna1d, Cacng4, Cald1, Cat, Ccdc91, Cdk5rap2, Chst3, Clip4, Ets1, Ezr, Fat1, Filip1l, Flt1, Hsd11b1, Igf2bp1, Iqsec3, Kndc1, Lamc1, Lrch1, Myh11, Pde1c, Plcg2, Prdm1, Prkch, Pvr, Rcbtb1, Rnd3, Sbk1, Sdc4, Spry4, Tns3, Tph2, Tshr*	MDD
Stress-resilient 3 and 6-month gain +LFC	184	*Aadat, Amph, Cryz, Gap43, Hyal2, Mocs1, Mpo, Mtg1, Pon1, Popdc2, Prkar1b, Ptprf, Rxra, Scarb2, Syt17, Zfyve28*	Anion binding
		*Acss1, Actr3b, Ak5, Ak7, Atp9a, Camk2b, Cdkl4, Chd5, Clcn4-2, Fgr, Galk1, Gck, Gucy2c, Hunk, Iars2, Iqca, Mcm2, Myh6, Myo15, Nek7, Nlrc3, Nlrp1a, Nubpl, Pfkm, Prkce, Prkg2, Sgk3, Slfn9, Trpm4, Ulk4*	ATP binding
		*Amph, Bcap29, Fez2, Inpp5a, Mvb12b, Nek7, Nkx6-3, Sgk3, Tmem259, Tpd52*	MDD

STRING analysis on the 5mC regions that are shared between 3- and 6-month susceptible or resilient animals also showed either a concomitant increase (+LFC) or decrease (–LFC) in 5mC and gene expression. We then compared our gene list to a published list of MDD genes. The table is a continuation of Fig. 3.

**Table 4.** Gene list of annotated DhMRs shared between 3- and 6-month animals.

	No. of genes in data set	Genes in network	Biological process
Stress-susceptible 3 and 6-month loss –LFC	269	*Bag3, Cbl, Csnk1d, Daam1, Dag1, Dagla, Dzip1, Fam53b, Fat4, Fgf7, Grin2d, Hnf1a, Ift172, Il17rd, Itga2, Jag2, Lrp6, Mark1, Nedd9, Nlk, Pkd1, Plxna1, Ptk2, Rap2a, Sel1l, Sema4d, Sort1, Sufu, Tgfbr2, Tle3, Tnks, Usp15*	Cell surface receptor signaling pathway
		*Aak1, Adarb1, Adat3, Asxl1, Brap, Camk1d, Cand1, Ccnd3, Cdk19, Csnk1d, Cttnbp2nl, Dcst1, Dyrk1a, Egln1, Epc1, Galnt2, Hdac4, Hnf1a, Kansl1, Kdm4a, Kdm5b, Kdm6b, Klhl21, Klhl24, Klhl3, Klhl42, Map3k2, Mark1, Mast4, Morc3, Nlk, Pkd1, Ptpn1, Ptprb, Pxk, Setd7, Sik1, Srpk1, St3gal2, St6gal1, Stat5b, Stk4, Tmtc2, Tnks, Trrap, Ube2h, Ubr5, Usp10, Usp15, Usp19, Usp24, Yeats2*	Macromolecule modification
		*Adra1b, Akap12, Dag1, Dstyk, Dusp4, Ece1, Fn1, Hmgcr, Iqgap1, Map3k14, Map3k2, Map3k5, Mfhas1, Mid1, Mink1, Nod1, Pik3r5, Ptpn1, Rap2a, Rara, Sema4c, Spred3, Timp3, Trib2, Wwc1*	MAPK and ERK1/2 signaling cascade
		*Ak4, Ankrd27, Arhgef10, Atrn, Avil, Bag3, Bhlhe40, Btbd3, Cic, Clmn, Dagla, Dock10, Dock7, Dpysl3, Fat4, Gnpat, Hspa5, Ift172, Jag2, Kif5a, Lrp6, Ncan, Omp, Sdk1, Serinc5, Slc38a2, Sox5, Sufu, Tmem108, Zfp365, Zfp609*	Nervous system
		*Aak1, Agfg1, Ank1, Ankrd6, Arl10, Asxl1, Bag3, Cadps, Camk1d, Ccnd2, Cdk19, Clmn, Dcp2, Dstyk, Dusp4, Dzip1, Entpd1, Fbxo28, Flnb, Galnt2, Heg1, Hmgcr, Hspa5, Iqgap1, Itga2, Itpkb, Kank1, Kif5a, Klhl24, Map3k, Mcf2l, Mink1, Ncan, Nedd9, Osbpl3, Pde4a, Pdzd2, Pfkfb3, Plxna1, Ptpn1, Sik1, Slc4a4, Sox5, St6gal1, Synpo2, Trib2*	MDD
Stress-susceptible 3 and 6-month gain +LFC	144	*Ascc1, Bag5, Cct8, Cd1d1, Celf2, Celf4, Corin, Cotl1, Ctnnbip1, Ddx24, Dnajc8, Eefsec, Enoph1, Fam192a, Hsf5, Insm2, Kcnk2, Keap1, Lpin1, Lrpprc, Map2k5, Mark2, Mef2c, Mgmt, Ndufab1, Nmnat3, Nosip, Nsmce2, Nudt3, Pard6g, Pdlim7, Ppargc1b, Ppp2cb, Prkag2, Pwwp2a, Qsox2, Rab3ip, Rbm25, Rcor2, Sap30bp, Smarca2, Smyd2, Spdef, Tacc2, Taf3, Taf9, Tcf7l1, Terf2, Tjp3, Tkt, Trappc12, Txk, Usp42, Vrk1, Wdr70, Xab2, Xrcc5, Ybx3, Yeats4, Zbtb8a, Zc3h10, Zfp30, Zfp536*	Nuclear Localization
		*Adora1, Bloc1s5, Caml, Cspg5, Dctn6, Emid1, Fam110b, Fars2, Fpgs, H2-DMa, Hs3st1, Idh2, Lgmn, Lipc, Mthfd1l, Oca2, Pcsk5, Pde9a, Rab4a, Rap1gap, Rer1, Rffl, Scnn1a, Slc37a1, Slc38a9, Slc48a1, Stambpl1, Tbxas1, Trf, Wnt3, Wnt4, Zdhhc20*	Membrane-bound organelles
		*Bckdhb, Celf2, Dtnb, Emid1, Fbxo9, Kiz, Map2k5, Mark2, Nudt3, Oca2, Ppargc1b, Prkag2, Scnn1a, Slc38a9, Smyd2, Tspan3, Vrk1, Yeats4, Zc3h10*	MDD
Stress-resilient 3 and 6-month loss –LFC	228	*Arhgef2, Bai1, Baiap2, Ccdc88c, Cntln, Crocc, Ctnna1, Daam2, Fes, Fgf7, Flii, Flnb, Kcnn2, Kif26b, Kif5a, Map2, Map3k1, Mark2, Mast3, Mib2, Mtus1, Myh9, Myo18a, Nexn, Pstpip2, Pxk, Rcsd1, Sorbs1, Specc1l, Ss18, Triobp, Wasf2*	Cytoskeleton organization
		*2510009E07Rik, Arhgef2, Bag3, Bag6, Bai1, Baiap2, Baiap3, Bend6, Casz1, Cln5, Cpeb1, Crtc1, Csk, Ctnna1, Daam2, Fes, Il6st, Jag2, Kcnj10, Kif5a, Lsr, Map2, Mark2, Megf8, Nav1, Nexn, Ppard, Prickle1, Rap1gap, Rara, Sall1, Sall3, Serinc5, Sh3gl3, Smad1, Snx3, Sorbs1, Sox13, Specc1l, Spg20, Sphk2, Tcf12, Tmeff1, Triobp, Tulp3, Zfp335, Zfp523, Zfp609*	Nervous system
		*Akap7, Ankrd6, Atg4c, Bag3, Best1, Brd4, Daam2, Dlgap4, Dzip1, Flnb, Hspbap1, Kank1, Kazn, Kif5a, Mark2, Myo18a, Nexn, Plekhh1, Pou2f2, Prkag2, Rsbn1l, Sh3gl3, Sik1, Slc36a1, Smad1, Sorbs1, Tmem120b*	MDD
Stress-resilient 3 and 6-month gain +LFC	204	*Abcd3, Adam3, Ap2a2, Atp5j2, Bbs9, Camk2g, Caml, Cdhr3, Clrn1, Cnih3, Corin, Daglb, Enpp2, Gpr157, Itm2b, Kl, Klhdc8b, Krt1, Laptm4a, Lpar5, Lpin2, Mios, Mtmr3, Ostf1, Pam, Pcsk4, Pcsk5, Pde2a, Pdgfra, Ptprr, Rnf139, Rtkn2, Sfxn1, Slc29a4, Slc37a2, Slc41a3, Slit1, Sntg2, Sptlc2, St6galnac5, Ston1, Suclg1, Tbxa2r, Tmprss6, Txk, Vps4a*	Cellular membrane
		*Alg14, Bcl2l1, Cbx3, Ctnnb1, Dpy19l1, Ipo11, Lrpprc, Prkg2, Rap1gap2, Sec13, Senp2, Unc50, Vapa*	Nuclear membrane
		*Bcl2l1, Cacng8, Camk2b, Clstn3, Cntnap4, Ctnnb1, Dlgap3, Grid1, Grin2b, Kcnj4, Lrrtm4, Lypd6, Scamp5, Sh3gl2, Syngr1*	Synapse
		*Aagab, Btbd9, Dpy19l1, Fez1, Fstl4, Hnrnpdl, Itm2b, Klhl32, Macrod1, Map10, Mrps6, Mtmr3, Ncald, Nek7, Nlrx1, Pank2, Pdgfra, Pkig, Ptprr, Rap1gap2, Sgk3, Sh3gl2, Sptlc2, Syngr1, Tcerg1l*	MDD

STRING analysis on the 5hmC regions that are shared between 3- and 6-month susceptible or resilient animals also showed either a concomitant increase (+LFC) or decrease (–LFC) in 5hmC and gene expression. We then compared our gene list to a published list of MDD genes. The table is a continuation of Fig. 3.

We identified 341 3- and 6-month common susceptible genes that concomitantly decreased in 5mC and gene expression that were found to have functions in the heart/circulatory system, ion transport, nervous system development, and catalytic activity (Table 3). We observed a large number of genes involved in the management of calcium release associated with synaptic neurotransmission and other genes linked to synaptic function. Genes that regulate calcium-induced neuro-vasculature blood flow, blood brain barrier (BBB) composition, and vascular growth signals were also observed. Encouragingly, we uncovered several genes linked to nutrient-sensing and glucose transport, further supporting our findings that disruption of glucose and energy homeostasis in chronically stressed animals could promote stress susceptibility. With respect to stress and anxiety, we identified *Glp1r,* which directly interacts with the HPA axis to trigger the secretion of glucocorticoids (Ghosal *et al*. 2013) and *Gpr3* which modulates anxiety- and depressive-related behaviors by regulating monoaminergic neurotransmitters and their metabolites (Valverde *et al*. 2009). Of the 54 genes found to overlap with the published MDD genes, 32 functions in the above biological process, supporting the idea that compromised brain health and energy homeostasis could be key contributors to stress susceptibility. In the 5mC concomitantly increasing susceptible group, 61 genes were found to function in the endocrine system (Table 3). Genes pertaining to lipid biosynthesis, metabolism, homeostasis, peripheral immune system activation, and male reproduction, were identified. Importantly, all of these processes are highly interconnected with HPA-axis activation during adaptive stress response (Tsigos *et al*. 2020).

5mC concomitantly decreasing genes in our common resilient category were found to be involved in pathways regulating heart/circulatory development, immune response, and various signaling pathways such as PI3k-Akt, MAPK, and ERK1/2 (Table 3). Although most of the genes in the heart/circulatory development pathway function primarily in muscle and heart tissue, there are several interesting genes that could function in the vasculature system of the brain. For example, *Agtr1a* is the receptor for angiotensin ll, a vasoconstrictor that regulates blood pressure. Recently, it was determined that angiotensin II induced hypertension increased BBB permeability and that Agtr1a receptors present in brain endothelial cells of the BBB increase its permeability (Santisteban *et al*. 2020). In the immune response category, we predominately observed genes that regulate inflammation and cytokine production. Traditionally, chronic stress-induced neuroinflammation compromises the integrity of the BBB which in turn has adverse effects on the neuro-vasculature architecture that supplies the brain with blood and other nutrients. Our data insinuates that the shared resilient regions between 3- and 6-month animals could repress these pathways through the loss of 5mC, proposing a neuroprotective role. In the overlapped MDD genes, we found several genes with various nervous system roles such as the adenosine deaminase, *Adarb2*, which can edit the serotonin receptor 2C mRNA and drastically affect its affinity for serotonin binding (Di Narzo *et al*. 2014) and the NADPH-dependent enzyme *Hsd11b1*, which converts cortisone to the stress hormone cortisol (Tomlinson *et al*. 2004). On the other hand, in resilient animals when 5mC and gene expressed concomitantly increased, genes involved in anion and ATP binding were identified (Table 3). We found several genes involved in neuroprotection, GABAergic signaling, and anxiety regulation. Overall, these findings lay out a mechanism whereby stress-induced alterations to 5mC in resilient animals exert a neuroprotective effect through the inhibition of neuroinflammatory and blood vessel constriction and the promotion of genes thought to protect against anxiety.

Concerning 5hmC in susceptible animals, 269 3- and 6-month common concomitantly decreasing genes were found to function in processes ranging from signaling cascades to the nervous system. More specifically, processes such as oligoprogenitor cell differentiation, BBB permeability, neuroinflammatory response, and the trafficking of glutamate receptors were observed (Table 4). One gene of interest includes *Dag1*, which is speculated to function in BBB integrity due to its expression in astrocytic feet that surround blood vessels and in endothelial cells that make up the BBB (Zaccaria *et al*. 2001). Importantly, many genes within all the clusters were involved in depression and anxiety-related behaviors in mice. For example, *Adarb1* targets the pre-mRNA of the GABA_A_ receptor (Ohlson *et al*. 2007) and serotonin receptor (Burns *et al*. 1997) for A-to-I editing. Moreover, 12 genes, 9 of which overlap with the published MDD genes, are implicated in Alzheimer's disease (AD). Also in the MDD overlap list is *Sik1,* a critical regulator of the corticotrophin-releasing hormone, a major hormone in the HPA-axis (Wang *et al*. 2022). One hundred and forty-four concomitantly increasing 5hmC genes in susceptible animals localized to the nucleus and membrane-bound organelles (Table 4). Among them, many genes function as epigenetic regulators of the chromatin and histone landscape, in microglia activation and neuroinflammation, as transporters across the BBB, or are linked to depression-related behaviors in mice. We again observed numerous genes associated with AD pathology, specifically the A -plaques, neurofibrillary tangles, and neurodegeneration. Nineteen of our genes overlapped with the published MDD data set and were found to function processes such as dopaminergic health, microglia activation, BBB permeability, and energy/metabolic β. Collectively, alterations to the 5hmC landscape in stress-susceptible animals appear to be linked to the dysregulation of neuroprotective processes, that under normal conditions, reinforce BBB permeability, regulate glutamate receptor availability, and neuroinflammatory process. Interestingly, many of the genes were found to also be associated with AD, supporting the proposed theory that chronic stress is a risk factor for AD (Machado *et al*. 2014).

The 228 shared concomitantly decreasing 5hmC genes identified in resilient animals function in cytoskeleton organization and the nervous system (Table 4). Gene analysis from both clusters demonstrated that processes such as synaptic vesicle trafficking and transmission, microglia activation, BBB regulation, sphingolipid metabolism, and Notch/Wnt signaling regulation could be disrupted. In the MDD cluster, we identified: *Brd4* which is associated with neuroinflammation, anxiety-like behavior, and impaired memory (Wang *et al*. 2021), the postsynaptic scaffolding protein, *Dlgap4*, which forms a protein complex to regulate glutamatergic synapses (Kim *et al*. 1997) and *Slc36a1,* which has been proposed to be a putative biomarker for patients who experience their first depressive episode after the age of 50 (known as late-onset MDD) (Miyata *et al*. 2016). Regarding the 204 genes displaying a concomitant increase in 5hmC and gene expression, their gene products were found to localize to cellular or nuclear membranes and synapses (Table 4). Of interest, the gene encoding β -catenin, *Ctnnb1*, was found and has previously been shown the regulate stress resilience in the nucleus accumbens (Dias *et al*. 2014). Other synaptic localizing genes include those that encode ion channels and synaptic vesicle endocytosis machinery. Notably, there appeared to be a significant bias toward GABAergic and glutamatergic synapse function and composition compared to other synapses. A serendipitous finding was the number of genes implicated in the regulation of adult neurogenesis and adult OPCs. Perturbations to both processes have been observed to influence anxiety and mood disorders (Pepper *et al*. 2018; Jones *et al*. 2022). Our findings imply that the loss of intragenic 5hmC in resilient animals appears to encourage the resilient phenotype by repressing neurodegenerative processes to some extent; suggesting that resilient animals are not impervious to the negative consequences of chronic stress. On the other hand, the gain of 5hmC could promote adult neuro- and glio-genesis.

### Acute social defeat induced epigenetic alterations that prime chronic stress response

Given the acute stress response observed during the first 2 days of CSDS, we modified the paradigm to recapitulate a short-term, ASDS. ASDS animals underwent 2 days of social defeat followed by social interaction testing (Supplementary Fig. 4a). Nest building and food consumption were measured daily, and as expected, on days 3 and 4 when the social defeat occurred, a reduction in nest quality and food consumption was observed (Supplementary Fig. 4b and c). There was a significant decrease in social interaction (Supplementary Fig. 4d) and sucrose consumption (Supplementary Fig. 4e), indicating that 2 days of stress is sufficient to induce depressive-like behavior. Importantly, a significant increase in corticosterone levels was only observed immediately following the final defeat (Supplementary Fig. 4f) and not 36 h after when tissues were collected (Supplementary Fig. 4g). This corresponds to the negative feedback mechanism of the HPA-axis, allowing for the maintenance of relatively low concentrations of corticosterone in the bloodstream. With this in mind, the resulting DNA modification profiles are more likely to represent a “recovery” period indicative of the lingering effects and not the immediate consequence of corticosterone.

**Fig. 4.**
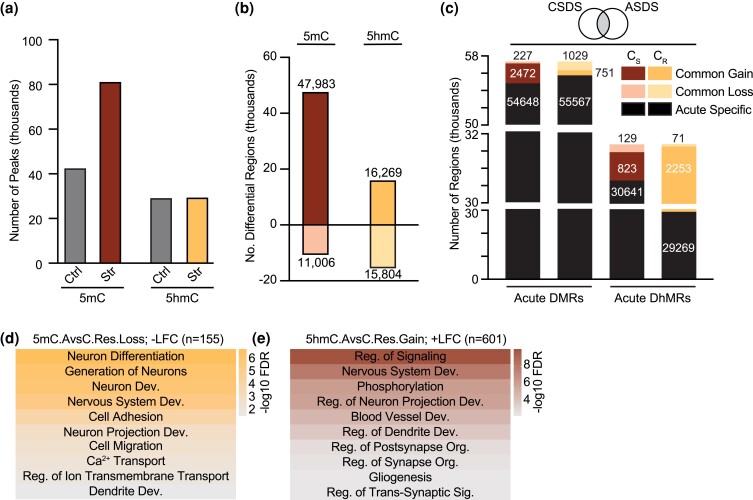
Characterization of DMRs and DhMRs from ASDS. a) Number of 5mC and 5hmC peaks identified in control and acutely stressed animals. b) Number of gained and lost 5mC and 5hmC peaks. GO analysis of the gain or loss of DMRs and DhMRs is in Table 5. c) Overlap between ASDS DMRs or DhMRs with CSDS susceptible or resilient specific DMRs or DhMRs. d and e) Representative GO analysis of the common genes identified between ASDS stressed and CSDS resilient mice that either concomitantly decreased in 5mC and gene expression (d. loss; –LFC) or concomitantly increased in 5hmC and gene expression (e. Gain; +LFC). Color scale represents -log_10_ FDR. GO analysis for the remaining 5mC and 5hmC analysis are in Table 6. Abbreviations: C_S_: chronic susceptible-specific, C_R_: chronic resilient specific, Reg.: regulation, Dev.: development, Org.: organization, Sig.: signaling.

Initial analysis of the 5mC and 5hmC peaks revealed a specific increase in 5mC peaks, and not 5hmC, in response to acute stress (Fig. 4a). In support of this, nearly 3.5-times more gained DMRs compared to lost were reported and the DhMRs were equivalently affected (Fig. 4b). Enrichment analysis uncovered inversely correlated 5mC profiles in every intragenic region except exons, whereas 5hmC enrichment was unaltered from the expected distribution in acute vs control samples (Supplementary Fig. 4h and i). These data suggest that the early accumulation of 5mC in response to acute stress could be a driving factor in the re-establishment of adaptive homeostasis whereas 5hmC could be more stable and less impacted by stress. ASDS-specific DMRs were identified in the neuronal calcium-binding protein, *Necab2*, the high-affinity adenosine receptor, *Adora2*, and the dopamine receptor subunit, *Drd2* (Supplementary Fig. 4j–l). Notably, in the 3-month CSDS analysis, we also observed a resilient specific loss of 5mC in *Drd2*, suggesting reduced methylation of *Drd2* could be an early indicator of stress resilience.

After the differential regions were correlated with RNA-seq data, GO analysis was used to determine the biological processes affected by ASDS (Table 5). Concomitantly increasing 5mC genes are involved in modulating a cell's response to stress. This suggests that a drastic or sudden change in activity, such as alterations in phosphorylation-mediated signaling, cell death, or the transport of ions, could contribute to the re-establishment of a homeostatic state. There is also the potential activation of a humoral immune response, which could be indicative of the immune system “preparing” to respond to insults such as the wounding that occurs during the social defeat. Although no significant biological processes were found in the concomitantly decreasing 5mC gene list (Table 5), more genes involved in signaling response to stress, like through cell surface receptors, were observed. This is in contrast to “fight-or-flight” related responses (reproduction, heart rate, muscle contraction, and blood pressure regulation) stimulated through the release of corticosterone where less genes were observed. Genes containing regions that concomitantly gained 5hmC function in metabolic and signaling processes such as glycerolipid metabolism (Table 5). In the brain, lipids facilitate synaptic membrane composition, energy homeostasis, and intracellular signaling process which could be regulating the protein localization or modification processes found in our data. Surprisingly, we also observed processes like molting and hair follicle and epidermis development. This could be a result of stress-induced shedding or hair and skin regrowth after injury. In the 5hmC concomitantly decreasing genes (Table 5), processes that regulate RNA transcription and biosynthetic processes, as well as histone modifying mechanisms were observed. This suggests some degree of dysregulation in gene expression. Downregulation of catecholamine secretion, which regulates “fight-or-flight” responses was also observed. Given that catecholamines can stimulate an immune response (Madden *et al*. 1995), a decrease in their secretion could also explain the observed decrease in immune response and Nuclear Factor Kappa Beta (NF-kB) signaling in our data. Overall, the analysis suggests that after acute stress and when corticosterone levels return to baseline, the reminiscent changes in 5mC appear to regulate cellular signals at the global scale, where 5hmC seems to be “fine-tuning” or perhaps orchestrating these global signals into more precise stress-responding pathways.

**Table 5.** Biological processes corresponding to the ASDS gained/lost DMRs and DhMRs.

DNA modification (numb genes gain/loss)	Gain; +LFC (−log 10 FDR)	Loss; –LFC (−log 10 FDR or genes)
5mC (*n* = 2,914/248)	Nervous System Dev. (9.5) Phosphorylation (8.3) Ion Transport (7.6) Homeostatic Process (7.5) Chemical Homeostasis (6.6) Reg. of Response to Stress (4.7) Reg. of Cell Death (4.3) Vesicle-Mediated Transport (4.2) Humoral Immune Response (4.1) Response to Wounding (4.1) Vasculature Dev. (3.9)	*Signal Transduction (55 genes)* *Response to Stress (27)* *Cell Surface Receptor Signaling (23)* *Nervous System Dev. (20)* *Immune Response (15)* *Reproductive Processes (15)* *Reg. of Heart Rate (2)* *Muscle Contraction (1)* *Blood Pressure Reg. (0)*
5hmC (*n* = 963/900)	Metabolic Process (9.8) Reg. of Signaling (8.8) Protein Localization (5.2) Reg. of Response to Stimulus (4.8) Protein Modification Process (4.6) Glycerolipid Metabolic Process (3.8) Nervous System Process (3.7) Hair Follicle Dev. (3.0) Respiratory System Dev. (2.9) Molting Cycle Process (2.8) Skin Epidermis Dev. (2.8) Response to Hormone (2.1)	+Reg. of RNA Biosynthetic Process (2.8) +Reg. of DNA-Templated Transcription (2.8) Reg. of Signaling (2.0) Macromolecule Modification (2.0) Reg. of Transcription by RNA pol ll (1.6) Reg. of Catecholamine Secretion (1.4) *-Reg. of MAPK Cascade (1.2)* *Histone Modification (1.2)* *Vascular Process in Circulatory System (0.9)* *Immune Response (0.9)* *NIK/NF-kappa* β*β* *Signaling (0.8)*

GO analysis of the genes from acutely stressed animals that either concomitantly increased in 5mC/5hmC and gene expression (Gain; +LFC) or concomitantly decreased in 5mC/5hmC and gene expression (Loss; –LFC). *n* = number of concomitantly increase/decrease genes. Italicized terms indicate those processes found not to be significant. Table corresponds to Fig. 4.

We next wanted to identify common concomitant genes between ASDS and CSDS to identify differential regions that could be indicative of a “priming” for stress susceptibility or resilience. To accomplish this, ASDS DMRs and DhMRs were overlapped with 3-month susceptible-specific or resilient-specific DMRs and DhMRs, respectively (Fig. 4c). Those common 2,472 regions annotated to 420 concomitant genes between ASDS gained DMRs and CSDS susceptible-specific gained DMRs. Furthermore, they are involved in processes that regulate neurovascular response to stress and cell–cell signaling likely mediated through cell junction, adhesion, and extracellular matrix mechanisms (Table 6). On the other hand, those susceptible genes share a common loss of 5mC function in various synaptic processes. For example, both *Farp1* and *Ptpro* promote synapse formation (Cheadle and Biederer 2012; Jiang *et al*. 2017) while *Mertk* helps mediate astrocyte-driven phagocytosis of synapses (Chung *et al*. 2013). In resilient animals, 136 genes were annotated from the 751 regions shared between ASDS and CSDS resilient-specific gain DMRs (Table 6). These genes function in adult neurogenesis, microglia-induced innate immune responses, BBB permeability, cell adhesion, and glutamate and glucose transport. Of interest, the *Dagla* gene encodes an enzyme that produces one of the main endocannabinoids in the adult brain and when *Dagla* expression is lost, animals develop anxiety and depressive-like behavior in mice (Jenniches *et al*. 2016). Those common resilient 5mC loss genes function in ion transport as well as neuron-specific processes like differentiation, generation, and projection (Fig. 4d).

**Table 6.** Identification of genes that could act as “primers” for stress response.

ASDS	CSDS specific file	No. of genes in data set	Biological process (−log10 FDR) or genes
Stress gain 5mC	Sus. gain 5mC	420	Circulatory System (4.0) Blood Vessel Morphogenesis (3.1) Vasculature Development (3.1) Response to Wounding (2.5) Cell Junction Organization (2.3) Enzyme-Linked Receptor Protein Signaling (2.2) Extracellular Matrix Organization (1.9) Nervous System Development (1.7) Cell Adhesion (1.7) +Regulation of Catalytic Activity (1.5) Synapse Organization (1.5)
Stress loss 5mC	Sus. loss 5mC	41	0610043K17Rik*, 1700010I14Rik, 4930539M17Rik, Adam2, Amotl1, Ankrd55, Cacna2d3, Cpped1, Crnde, Dnah8, Ebf1, Ephx2, Farp1, Gabrg3, Gm10635, Grm8, Itga2, Itgad, Mertk, Myo3a, Optc, Osbpl9, Pan2, Pard3, Pcdh15, Pde9a, Pou6f2, Ppp1r1c, Prkce, Prmt3, Ptpro, Rec114, Sec24d, Smoc1, Sorcs3, Spon1, Srgap1, Stk32b, Trdmt1, Ush2a, Zfp521*
Stress gain 5mC	Res. gain 5mC	136	*A730036I17Rik, Abca4, Adamts17, Ankrd44, Arhgef10l, Arhgef3, Asap1, Atp9a, Atrnl1, Bcar3, Brca2, Cadm3, Camta1, Capn9, Catsperg1, Cdc42bpg, Col5a1, Creb5, D830013O20Rik, Dagla, Dapk2, Dcaf4, Disc1, Dock2, Dock5, Dock8, Dysf, E330021D16Rik, Efcab6, Erc2, Esrrb, Fam221b, Fancd2, Fcgbp, Fli1, Fto, Gdpd5, Gm6249, Gna15, Gng2, Gpr68, Grhl2, Grik3, Haao, Hebp1, Hs3st3b1, Hunk, Hydin, Kalrn, Kank2, Kcnb2, Kcnk10, Kif26a, Kit, Ksr2, Lama4, Lmna, Lnx1, Lrig3, Lrrc28, Ltbp2, Megf11, Mgmt, Micu1, Morc1, Mras, Mvb12b, Myo16, Myrip, Naaa, Nlrp1a, Npc1, Nuak1, Nubpl, Nudt12, Nxph1, Osbpl3, Pde6c, Pex5, Phldb3, Piezo2, Plxnc1, Ppl, Prdm16, Prex1, Prkaa1, Ptprf, Rfx8, Rgs3, Rhbdd1, Rnf144b, Rora, Rrp9, Scfd1, Scgn, Sdk2, Shc3, Shq1, Slc13a3, Slc1a7, Slc22a1, Slc45a1, Slit1, Smad3, Smyd3, Snx18, Snx8, Sohlh2, Sorcs2, Spag16, Spats2l, St3gal4, Ston2, Stox2, Susd1, Swap70, Syne2, Tanc1, Tecta, Tgfbr2, Tmem117, Tmem178b, Tnfaip8, Tns1, Tpcn2, Trappc11, Trim47, Trp63, Ttc23, Usp43, Wdr63, Wdr70, Wnt7b, Wwc2, Zbtb7c, Zc3h18*
Stress loss 5mC	Res. loss 5mC	155	Neuron Differentiation (6.8) Generation of Neurons (6.2) Neuron Development (6.0) Nervous System Development (5.4) Cell Adhesion (4.0) Neuron Projection Development (2.8) Cell Migration (2.6) Ca^2+^ Transport (2.2) Regulation of Ion Transmembrane Transport (2.0) Dendrite Development (1.6)
Stress gain 5hmC	Sus. gain 5hmC	261	−Regulation of Response to Stimulus (2.9) Regulation of Fat Cell Differentiation (2.7) Enzyme-Linked Receptor Protein Signaling (2.6) Homeostatic Process (2.4) Regulation of K^+^ Transmembrane Transport (2.0) Response to TGFβ (1.8) Neuron Projection Development (1.8) Blood Vessel Development (1.6) Ion Transport (1.5) Regulation of Smooth Muscle Cell Migration (1.4)
Stress loss 5hmC	Sus. loss 5hmC	53	*2410004B18Rik, Actn4, Acvr2b, Ahsa2, Alkbh2, Arhgap10, Arid1b, Arid2, Bcr, Chst11, Chst12, Cmip, Coro1c, Daam1, Ddah1, Dnm3, Eif3h, Eif4g3, Eml1, Ephb1, Farp1, Foxj2, Grap2, Katnal1, Lims2, Lrrc1, Lrrc42, Map4k4, Mapre2, Mtcl1, Mtus2, Myl12b, Prep, Prrc2c, Rest, Ring1, Rnf126, Rptor, Serac1, Sik3, Slc12a1, Slc16a7, Smarca2*, *Spata13, Spock1, Tenm3, Thrsp, Ubr5, Vangl1, Zdhhc14, Zdhhc18, Zfp462, Zfp865*
Stress gain 5hmC	Res. gain 5hmC	601	Regulation of Signaling (9.6) Nervous System Development (8.0) Phosphorylation (6.5) Regulation of Neuron Projection Development (6.3) Blood Vessel. Development (5.5) Regulation of Dendrite Development (4.7) Regulation of Post-Synapse Organization (3.9) Regulation of Synapse Organization (3.7) Glio-genesis (3.4) Regulation of Dendrite Morphogenesis (3.4)
Stress loss 5hmC	Res. loss 5hmC	26	*Atxn7l1, Bcdin3d, Camkmt, Chst11, Chsy1, Dst, Gna12, Lrrn1, Maml3, Map4k4, Matn4, Mfsd5, Msantd1, Park7, Picalm, Prkce, Ric8b, Rnf114, Rnpep, Scn8a, Sept8, Slc9a9, Soga1, Tet3, Zfp513, Zfp710*

GO and gene analysis of the overlapped genes found between ASDS DMRs or DhMRs and CSDS susceptible or resilient specific DMRs or DhMRs. Table corresponds to Fig. 4.

Common concomitant genes between ASDS gain DhMRs and CSDS susceptible-specific gain DhMRs (Table 6) function in signaling cascades mediated through enzyme-linked receptor proteins and/or the transport of ions across membranes in response to a stimulus to re-establish a homeostatic state. The regulation of fat cell differentiation could be indicative of an adaptive response to compensate for the increased energy demand due to stress. Other processes include the upregulated response to transforming growth factor-β(TGF-β) signaling, which in turn is known to activate blood vessel development. Importantly, acute and chronic insult are both known to significantly upregulate TGF-β signaling (Vivien and Ali 2006), corroborating our finding. Interestingly, susceptible genes that share a common loss of 5hmC and gene expression encode subunits of the SWItch/Sucrose Non-Fermentable (SWI-SNF) ATP-dependent chromatin remodeling complex or polycomb repressive complex 1 (Table 6). The ASDS gain DhMRs that overlapped with the CSDS resilient-specific gain DhMRs annotated to genes that are primarily involved in signaling through phosphorylation and the organization of dendritic synapses (Fig. 4e). There was also a significant enrichment for glio-genesis, supporting our findings from the CSDS resilient DhMRs that stress could be disrupting the maturation or differentiation of adult oligoprogenitor cells. Those genes found to be in common with CSDS resilient-specific DhMR loss, appear to function in energy homeostasis and have neuroprotective roles (Table 6). Other genes of interest include *Pkce* and *Tet3*, both of which have been affiliated with depression or anxiety-like behaviors (Antunes *et al*. 2021; Pandey *et al*. 2021). These results are surprising considering the loss of neuroprotective properties is contradictory to the expected stress-resilient phenotype. It is possible that these findings are specific to acute stress response and that animals primed to be resilient have the potential to rectify these processes.

In summary, our comparison of 5mC and 5hmC between ASDS and CSDS to identify putative epigenetic priming patterns for stress susceptibility or resilience was informative. In general, the DNA profiles across susceptible and resilient animals demonstrate that both groups are affected by the dysregulation of stress-responding pathways. Susceptible animals specifically repressed subunits of chromatin remodeling complexes, while resilient animals appear to promote neuro- and oligo-genesis.

### Longitudinal social defeat suggests that epigenetic memory may protect against chronic stress

**Fig. 5.**
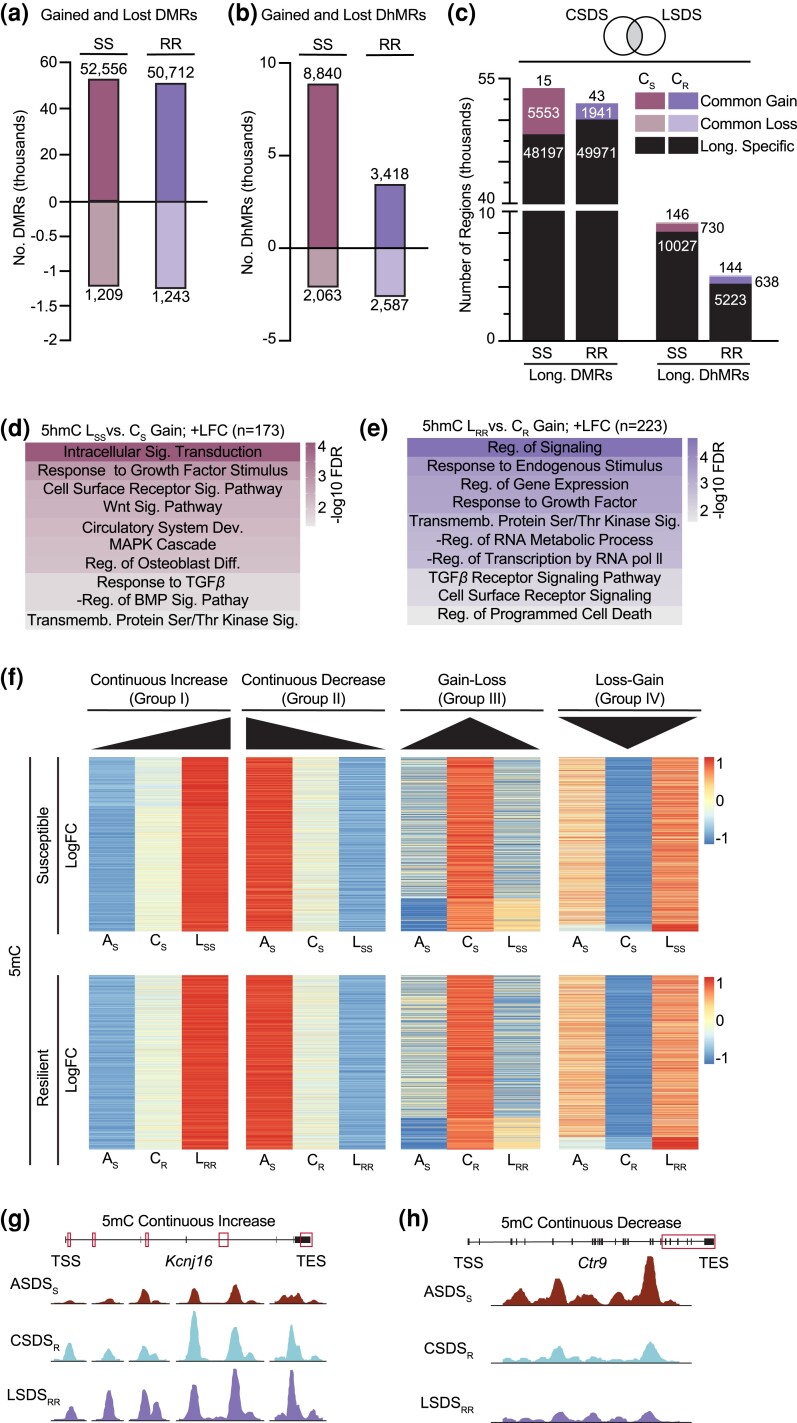
Characterization of DMRs and DhMRs from LSDS. a and b) Number of gained and lost 5mC (a) and 5hmC (b) peaks in longitudinally stressed animals. c) LSDS SS and RR gained or lost DMRs or DhMRs overlapped with CSDS susceptible or resilient specific DMRs or DhMRs. d and e) Representative GO analysis of the common genes identified between L_SS_ and CSDS susceptible (d) and L_RR_ and CSDS resilient (e) mice that concomitantly increased in 5hmC and gene expression (gain; +LFC). GO analysis for remaining 5mC and 5hmC analysis are in Table 7. f) Average normalized 5mC read count across ASDS, CSDS, and LSDS susceptible (top) or resilient (bottom) continuum with continual 5mC accumulation (Group I), depletion (Group II), or a return to baseline (Groups III and IV). g and h) Normalized 5mC counts at peak regions identified across ASDS, CSDS, and LSDS: *Kcnj16* (g), *Ctr9* (h). Abbreviations: SS: susceptible remained susceptible, RS: resilient to susceptible, RR: resilient remained resilient, C_S_: chronic susceptible-specific, C_R_: chronic resilient specific, Sig.: signaling, Reg.: regulation, TGF β: transforming growth factor-Betta, BMP: bone morphogenetic protein, Transmemb.: transmembrane, Ser/Thr: serine/threonine, TSS: transcription start site, TES: transcription termination site.

Previous work has demonstrated that CSDS has an enduring influence on social behavior (Sial *et al*. 2016); however, how this effect differs between susceptible and resilient stress responders is poorly understood. To model this behavior, we employed a longitudinal social defeat stress (LSDS) paradigm that incorporates an “incubation” period into the CSDS paradigm followed by a second re-exposure social interaction (Supplementary Fig. 5a). Animals were divided into 2 groups based on their behavior in the social interaction test: Animals who maintained susceptibility (SS) and those who remained stress-resilient (RR) (Supplementary Fig. 5b). Initial peak analysis showed that overall, re-exposure induced an increase in both 5mC and 5hmC peak number compared to controls (Supplementary Fig. 5c). Overall, 5mC peaks were specifically enriched in exon regions while 5hmC peaks were generally enriched in intragenic regions (Supplementary Figs. 5d, 5e). The number of gained and lost DMRs was approximately equal across all stress groups (Fig. 5a) whereas, for 5hmC, the SS animals have more than twice as many gained DhMRs compared to the RR animals (Fig. 5b). Our data suggests that re-exposure simulates acute stress, given that both ASDS and LSDS display a spike in 5mC peak number and gained DMRs compared to CSDS and in terms of the stress duration. Regarding 5hmC, although SS animals have over twice as many gained DhMRs compared to RR, when considered across all stress conditions (Figs. 2c, 4b, and 5b), 5hmC dynamics appear to be decreasing or displaying minimal alterations. This suggests once 5hmC is added or removed from a region, it functions as a stable epigenetic mark. To investigate how DNA modifications can modulate stress response after a re-exposure event, we applied the same overlapping method described in Fig. 4c to compare CSDS susceptible and resilient specific differential regions to LSDS SS (L_SS_) and RR (L_RR_) gained and lost differential regions (Fig. 5c). We identified 711 overlapping concomitant genes that gained 5mC between L_SS_ and CSDS susceptible DMRs (Table 7). Interestingly, these genes were enriched in numerous peripheral body systems associated with the sympathetic nervous system, suggesting a potential function in the signaling processes between these 2 networks. The regions that overlapped between L_SS_ and CSDS susceptible lost DMRs only annotated to the two genes *Prdm16* and *Zswim4*, whose functions in the adult brain are unclear (Table 7). On the other hand, the overlap of L_RR_ and CSDS resilient gained DMRs revealed 234 genes, where the only significant biological processes are neuromuscular process and intracellular signal transduction (Table 7). Despite their lack of statistical significance, the synaptic vesicle cycling and ionotropic glutamate receptor signaling processes could suggest an important function for glutamate receptor recycling homeostasis in developing stress resilience. Furthermore, the HPA-axis and circadian regulation are deeply interconnected in their efforts to regulate stress response. Very few genes overlapped between L_RR_ and CSDS resilient loss DMRs (Table 7). Among the genes identified, we observed the BBB transporter (*Abcc4*) (Warren *et al*. 2009), the neural tissue-specific chromatin remodeler (*Chd5*), and the myelinating protein (*Mopb*) (Holz *et al*. 1996).

**Table 7.** Biological processes and genes affected after stress re-exposure.

DNA modification	LSDS	CSDS specific file	RNA-seq	No. of genes in data set	Biological process (−log10 FDR) or genes
5mC	L_SS_ gain	Sus. gain	+LFC	711	System Development (12.2) Nervous System Development (6.4) Ear Development (5.9) Circulatory System Development (5.3) Tissue Development (5.3) Renal System Development (4.7) Kidney Development (4.6) Vasculature Development (3.8) Skeletal System Development (2.5) Respiratory System Development (2.5)
	L_SS_ loss	Sus. loss	−LFC	2	*Prdm16 and Zswim4*
	L_RR_ gain	Res. gain	+LFC	234	Neuromuscular Process (1.4) Intracellular Signal Transduction (1.4) *Synaptic Vesicle Cycle* *Circadian Regulation of Genes Expression* *Cilium Organization* *Ionotropic Glutamate Receptor Signaling Pathway*
	L_RR_ loss	Res. loss	−LFC	13	*Abcc4, Adprh, Chd5, Dennd3, Edar, Igf2bp2, Itpr3, Lamb2, Mobp, Ptprg, Rbm45, Rtn1, Sec14l5*
5hmC	L_SS_ gain	Sus. gain	+LFC	173	Intracellular Signal Transduction (4.1) Cellular Response to Growth Factor Stimulus (2.9) Cell Surface Receptor Signaling Pathway (2.5) Wnt Signaling Pathway (2.4) Circulatory System Development (2.3) MAPK Cascade (2.2) Response to TGF-β Stimulus (1.7) -Regulation of BMP Signaling Pathway (1.7) Transmemb. Receptor Protein Serine/Threonine Kinase Sig. (1.4)
	L_SS_ loss	Sus. loss	−LFC	113	*Afap1l1, Akt1, Aldh4a1, Arhgap23, Asap1, Asap2, Atp9a, Bmp2k, Ccdc6, Chst12, Clic4, Clip2, Cmip, Ctdspl, Ddx54, Dnah8, Eml1, Foxo3, Foxp1, Frmd4b, Fyn, Gatc, Gpr146, Grip1, Lmf1, Lnx2, Lrrc8d, Map4k4, Mast4, Mbd3, Mob3b, Mtcl1, Ncor2, Nfia, Nfkb1, Ntm, Pik3r1, Pkp4, Prrc2c, Ptpru, Rap1gds1, Sema6a, Sik1, Smc5, Sox5, Spata13, Spock1, Spred2, Stox2, Tmcc2, Tmem63b, Trio, Trub1, Upp2, Zbtb43, Zfp462, Zfp532, Zfp768, Zswim6*
	L_RR_ gain	Res. gain	+LFC	223	Regulation of Signaling (4.7) Response to Endogenous Stimulus (3.6) Regulation of Gene Expression (3.4) Response to Growth Factor (3.3) Transmemb. Receptor Protein Serine/Threonine Kinase Sig. (2.9) -Regulation of RNA Metabolic Process (2.9) -Regulation of Transcription by RNA pol ll (2.7) TGFβ Receptor Signaling Pathway (2.0) Cell Surface Receptor Signaling Pathway (2.0) Regulation of Programed Cell Death (1.6)
	L_RR_ loss	Res. loss	−LFC	67	*2810013P06Rik, Abhd2, Acot8, Acvr1, Ankrd11, Apc2, Arhgef10, Arhgef2, Arid3a, Atp13a2, Atp9a, Atxn1, Bcdin3d, Best1, Camsap1, Cebpg, Chd7, Cmip, Col27a1, Csrnp1, Ctdspl, Dnajb3, Dock9, Eif4g3, Elfn2, Eml1, Fam110b, Farp1, Foxo3, Gna12, Gramd1a, Iffo2, Il4i1, Itpk1, Kank2, Klf3, Ksr1, Lrfn3, Map3k1, Map3k4, Mcu, Mical1, Micu1, Mmp15, Nphp4, Pced1b, Pkp4, Ppm1b, Prkce, Rapgef3, Rara, Rbpms, Rusc2, Ski, Slc25a51, Slc38a10, Srgap1, St3gal3, Tceanc2, Tet3, Tmem184b, Vgll4, Wnk2, Zbtb5, Zfhx2, Zfp513, Zswim6*

GO and gene analysis of LSDS DMRs and DhMRs overlapped with CSDS susceptible or resilient specific DMRs or DhMRs. Table corresponds to Fig. 5.

We identified 173 overlapping concomitant genes that gained 5hmC between L_SS_ and CSDS-susceptible DhMRs (Fig. 5d and Table 7). There is a strong enrichment for signaling cascades such as Wnt, MAPK, TGF-β and BMP signaling, which all utilize cell surface receptors and serine/threonine kinase signaling to facilitate their signal transduction. Importantly, these signaling cascades function in the brain and have broad impacts on how the brain responds to stress. Regarding the overlapping L_SS_ and CSDS susceptible lost DhMRs, 113 genes were identified and preferentially function in the nervous/neurovascular systems and signaling pathways (Table 7). Deficiency in the zinc finger *Zfp462* has recently been found to cause anxiety-like behavior (Wang *et al*. 2017) and could be a critical stress susceptibility gene. Similar to the gained DhMRs shared between L_SS_ and CSDS susceptible animals, the shared gained DhMRs between the L_RR_ and CSDS resilient animals also annotated to genes involved in cellular signaling utilizing cell surface receptors and serine/threonine kinase (Fig. 5e and Table 7). In addition, processes that regulate gene expression, potential through transcription, and RNA metabolic processes were also observed. Positive and negative regulation of cellular signaling are likely ubiquitous consequences of stress and are not necessarily distinguishing characteristics of stress susceptibility or resilience. However, our data hints at a possible mechanism whereby the processing of mRNA transcripts could be contributing to the diverging stress response phenotypes. Only 67 genes overlapped between L_RR_ and CSDS resilient loss DhMRs (Table 7). From these genes, we identified chromatin remodelers and transcription factors that function in various components of adult neurogenesis such as inducing adult neural stem cell proliferation (*Ankrd11*) (Gallagher *et al*. 2015), differentiation (*Chd7*) (Feng *et al*. 2013) or quiescence (*Foxo3*) (Renault *et al*. 2009).

In summary, after stressor re-exposure, 5mC was found to regulate the brain's communication network with peripheral organ systems in susceptible animals. For resilient animals, upregulation of 5mC is linked to the putative recycling of glutamate receptors and promotes HPA—circadian synergism. With respect to 5hmC, both susceptible and resilient animals express an upregulation of intracellular signaling cascades in response to stress. For susceptible animals, these signals could be contributing to the downregulation of nervous/neurovascular processes, whereas in resilient animals, the mRNA transcripts downstream of these signals could be undergoing various metabolic processes affecting gene expression.

### 5mC and 5hmC dynamics across the ASDS-CSDS-LSDS time course

To investigate stress-induced patterns that expand across the acute, chronic, and longitudinal (ASDS-CSDS-LSDS) time course, we examined DMRs and DhMRs that either continually increased or decreased (Fig. 5h and Supplementary Fig. 5f, Groups 1 and 2, respectively) or returned to baseline (Figs. 5h and Supplementary Fig. 5f, Groups 3 and 4, respectively). Given that the handling conditions and time between defeats, SI, and tissue collection were constant across each social defeat variation, the three conditions are suitable for a linear comparison to estimate a stress-dependent epigenetic timeline. Only those genes whose expression pattern matched the 5mC/5hmC pattern (i.e. both 5mC and gene expression increased between ASDS and CSDS and decreased between CSDS and LSDS) were considered (Supplementary Fig. 5g). Furthermore, we prioritized those genes that showed the most drastic pattern fluctuations for further discussion.

Genes that continuously increase or decrease in either DNA modification across the ASDS-CSDS-LSDS time course are likely to function as major contributors to stress response. For example, genes found along the depression susceptible course (A_S_-C_S_-L_SS_) would be of great interest, because they are most likely to impact stress-induced depression. In the susceptible 5mC continuously increasing group, we identified 334 genes (Fig. 5f, Group 1; top). This list contained several ion channels such as *Kcnj16* (Fig. 5g) as well as pyrimidine metabolizing and anxiety-related genes. On the other hand, 107 genes were observed to continuously decrease in 5mC (Fig. 5f, Group 2; top). Of those genes, *Ctr9* and *St6galnac2* expressed the most significant decreases. *Ctr9* (Fig. 5h) functions in a protein complex that traffics dopamine transporters to the plasma membrane (De Gois *et al*. 2015). The sialyltransferase enzyme, *St6galnac2*, transfers sialic acid onto the sugar *N*-acetylgalactosamine on the cell surface (Schnaar *et al*. 2014). The brain contains the highest levels of sialic acids which regulate a variety of functions such as neuronal plasticity, the myelination of axons, myelin stability, and neuronal network remodeling (Rawal and Zhao 2021). These observations are consistent with the idea that chronic stress-induced neurodegeneration is tightly linked with depression susceptibility. Those genes whose 5mC and gene expression returned to baseline in susceptible animals represent genes that may be working to reduce the allostatic load induced by chronic stress (Fig. 5f, Group 3 and 4; top). We identified clusters that either displayed a gain–loss (29 genes) or loss–gained (2,698 genes) pattern. From the gain–loss cluster, one of the most dramatic changes was observed in *Sgk3*. *Sgk3* has been shown to mediate corticosterone-induced autophagic cell death in neural stem cells after chronic stress (Jung *et al*. 2020). In the loss–gain cluster, we observed a series of genes involved in hormone transport (*Ttr*), vitamin A metabolism (*Rbp2*, *Rbp7*, and *Stra6*), metal ion homeostasis (*Steap1*) and brain transporter proteins (*Kcne2*, *Slc4a5*, and *Aqp1*). Based on the data presented, epigenetic recovery is predominantly occurring in genes that function in very primal and highly conserved processes, but there is also evidence to suggest some degree of HPA-axis recovery in susceptible animals.

Peculiarly, for both the 5mC continually increasing and decreasing resilient clusters (A_S_-C_R_-L_RR_) (Fig. 5f, Group 1 and 2; bottom), there were minimal pattern fluctuations compared to the depression susceptibility model. In the continuously increasing cluster, we identified genes that participate in synapse morphology (*Cadm1* and *Ap3b1*), nonhomologous end joining of DNA double-strand breaks (*Xrcc5*), inflammation-induced cell death (*Irf2*) and brain cholesterol metabolism (*Ephx2*). In the continuously decreasing cluster, genes related to intracellular iron homeostasis (*Bdh2*) and neurotransmitter release (*P2rx2*) were observed. Given the minimal increase or decrease of 5mC in depression-resilient animals, this provides additional support to our prior observations: First, that 5mC is functioning as an acute stress response modulator and second, that the LSDS re-exposure stressor is more analogous to an acute stressor. Resilient genes that returned to baseline also appear to work in basal neurological processes essential for proper cellular and brain function, similar to the susceptible animals. Only 29 genes make up the 5mC gain–loss cluster (Fig. 5f, Group 3), but the most significantly altered genes function in important processes like DNA damage regulation during replication (*Rfc5*), synapse morphology (*Musk* and *Zdhhc2*) and cholesterol-mediated steroid production (*Atad3a*). The loss–gain cluster contains 2,385 genes (Fig. 5f, Group 4; bottom). Several genes localize to the microvessels of the brain (*Slc47a1* and *Enpep*) or function in different aspects of nervous system inflammation and immunity (*Pf4*, *Mamdc2*, and *Slc9b2*). Overall, these data suggest that despite their resilient phenotype, depression-resilient animals are not impervious to the negative physiological consequences of chronic stress. An unexpected finding was the recovery and prevention of DNA damage processes. Accordingly, the promotion of adult neurogenesis and DNA damage prevention may be the processes most responsible for easing the long-standing effects of chronic stress, to a certain degree in resilient individuals.

Regarding 5hmC, 31 genes were found to continuously increase in both 5hmC and gene expression in our depression susceptibility model (Supplementary Fig. 5f, Group 1; top). The *Qrfpr* receptor showed the most significant increase (nearly 11-fold) of all the genes and has recently been implicated in rodent feeding behavior and dietary fat intake (Primeaux *et al*. 2008). In the 5hmC continuously decreasing group (Supplementary Fig. 5f, Group 2; top), many of the genes (*Ifgbp7*, *Tspan18*, *Flt4*, and *Acvrl1*) are expressed in the endothelial cells that make up the brain's vasculature or the BBB (*Pecam1*). We also identified the circadian clock gene, *Per1*, as being one of the most repressed genes in our list. Of particular interest, the DNA modifying proteins Dnmt3a, Tet2, and Tet3 were found, suggesting that the loss or reduced expression of epigenetic machinery could be a key contributor to stress-induced depression. Our data demonstrate that the continual worsening of 5hmC dynamics in susceptible animals is associated with the development of a compromised neuro-vasculature system and BBB. These phenotypes are reminiscent of early AD pathology and further support chronic stress as a major risk factor for AD. Subsequently, many of the genes from both groups, such as *G3bp2, Grhl1, Flt4,* and *Pecam1*, have all been previously associated with AD (Giri *et al*. 2000; Taracha *et al*. 2018; Wolozin and Ivanov 2019; Mahoney *et al*. 2021). The genes that returned to baseline expression in the depression susceptible (Supplementary Fig. 5f, Groups 3 and 4; top) pathway work in diverse biological functions such as ion transport (*Scn1a*), lipid metabolism (*Cyb5r3*), neuro-vasculature (*Slc4a5* and *Mgp*), neuroinflammation regulation (*Adora2b*), and excitatory synapse formation (*Syndig1*). Interestingly, we again identified the Klotho gene (*Kl*), which we previously found in 3-month CSDS-susceptible animals. In the central nervous system, *Kl* is secreted into the CSF and acts as a circulating hormone allowing it to exert its biological functions (mediating the effects of nitric oxide, oxidative stress, inflammation, and calcium metabolism) over the brain (Gold *et al*. 2013). More importantly, dysregulation of *Kl* has also been to neuropsychiatric disorders including MDD (Wu *et al*. 2022).

In depression-resilient animals, 117 genes were found to continuously and concomitantly increase in 5hmC (Supplementary Fig. 5f, Group 1; bottom), and several of the most drastic increases were observed in Ada, *Hpse2*, *Smoc2*, and *Disc1*. Adenosine deaminases (*Ada*) have multiple functions, one of which is to protect the nervous system from high levels of adenosine that can occur when nervous system activity is enhanced like under conditions of chronic stress (Wardas 2002). Both *Hpse2* and *Smoc2* function in the positive regulation of heparin sulfate. Heparan sulfates account for 50–90% of the residues in glycosaminoglycan side chains on glycocalyx, which coats the endothelial cells of the BBB, and reinforces impermeability to peripheral circulating molecules (Yang *et al.* 2021). Generally, SNPs and mutations in *Disc1* are well-established contributor to neuropsychiatric diseases. In the context of promoting resilience to depression, the increase in *Disc1* transcription in response to chronic stress could reflect an effort to manage the demands of its expansive interactome. Of the 269 genes that continued to decrease in 5hmC in resilient animals (Supplementary Fig. 5f, Group 2; bottom), we identified two transporter proteins (*Slc2a8* and *Slc38a4*) that facilitate the transport of glucose and large amino acids across the BBB, respectively. Furthermore, 425 gain–loss and 729 loss–gain genes for the 5hmC return to baseline clusters (Supplementary Fig. 5f, Groups 3 and 4; bottom) were observed. In these groups, we observed the metalloproteinase (*Mmp21*) and neuroinflammatory modulators *Tlr6* and *Alox5*. Importantly, prolonged activation of MMPs in the brain could weaken the BBB, over activate neuroinflammatory pathways, and cause demyelination of neurons (Rempe *et al*. 2016). In addition, we found thymine DNA glyucosylase (*Tdg*) which functions in the base excision repair mechanism to regenerate an unmodified C in the DNA cytosine modification biochemical pathway (Kohli and Zhang 2013). In the depression susceptible model, we found a continual decrease in the DNA modifying proteins (Dnmt3a, Tet2, and Tet3) while resilient animals display TDG returning to baseline. These observations would imply a role for DNA modifying machinery in influencing stress-induced depression.

In conclusion, our analysis of 5mC and 5hmC alterations spanning across the acute, chronic, and longitudinal time course provides a systematic and linear comparison of how DNA covalent modifications are regulated in response to various forms of stress. This analysis has shed light on the molecular mechanisms of how critical stress response genes are epigenetically regulated. Our final gene list represents the most likely ones to influence stress response and offer potential therapeutic targets for MDD.

## Discussion

**Fig. 6.**
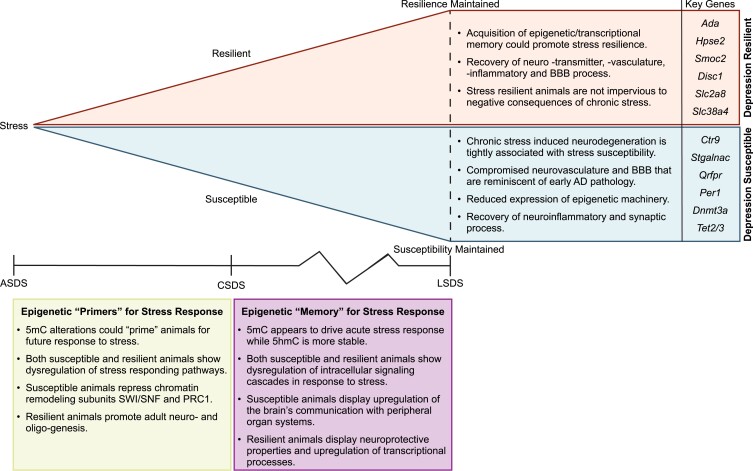
Summary of the 5mC and 5hmC dynamics across the ASDS-CSDS-LSDS time course. Diagram summarizing the key findings and putative key genes of the 5mC and 5hmC dynamics between ASDS and CSDS (bottom left box, Epigenetic "Primers" for Stress Response), CSDS and LSDS (bottom right box, Epigenetic "Memory" for Stress Response), and across the ASDS-CSDS-LSDS time course (top diagram).

In this study, we investigated how the DNA modifications 5mC and 5hmC could contribute to the individual differences in stress susceptibility and resilience by comparing their genome-wide profiles between animals that underwent various durations of social defeat: acute (short-term), chronic (persistent), and longitudinal (re-exposure). We characterized CSDS in 3-month-old and 6-month-old naive adult male mice. In 3-month animals, 5mC and 5hmC work in parallel and were not found to distinguish between stress-susceptible and resilient phenotypes, while 6-month animals displayed distinct 5mC and 5hmC enrichment patterns. Analyses of acute stress responses revealed a potential “priming” mechanism for 5mC that could foreshadow chronic stress response. The overlap of ASDS and CSDS differential regions revealed a potential 5hmC suppression of chromatin remodeling subunits in susceptible animals. On the other hand, resilient animals supported an epigenetic environment that promoted adult neuro- and glio-genesis. These findings insinuate that stress susceptibility may be predisposed if the epigenetic architecture is unable to rearrange during times of stress and that the promotion of adult neurogenesis may serve as a critical factor for priming stress resilience. Comparison between CSDS and LSDS differential regions suggest that the enduring effects of social defeat affect differential biological process between susceptible and resilient animals. Susceptible animals display an upregulation of peripheral organ systems, likely induced by the acute re-exposure stress, and repression of the nervous and neurovascular processes whereas resilient animals express an upregulation of metabolic processes of mRNA transcripts. Finally, the stress-induced 5mC and 5hmC fluctuations across the ASDS-CSDS-LSDS time course demonstrate that resilient animals are not impervious to the negative consequences of chronic stress and susceptible animals do display some degree of neuroinflammatory and synaptic processes recovery. These findings are summarized in Fig. 6.

Because constant conditions were maintained across all social defeat paradigms, we systematically compare fluctuations in 5mC and 5hmC dynamics across different durations of stress. We observed an accumulation of 5mC in response to acute stress that we hypothesized could be a driving factor in the re-establishment of adaptive homeostasis. Encouragingly, in our CSDS 5mC analysis, both susceptible and resilient animals show a significant decrease in their number of 5mC peaks and gained DMRs, compared to ASDS. However, susceptible animals gained nearly 1.5 times more DMRs compared to resilient. In addition, susceptible and resilient animals showed another significant increase in 5mC and gained DMRs in our re-exposure LSDS analysis. Accordingly, this suggests that the “re-exposure” stressor simulates acute stress, resulting in a sizeable 5mC increase that was also observed in ASDS. This is further supported by the minimal continual increase or decrease of 5mC in depression-resilient animals observed over the ASDS-CSDS-LSDS time course. Taken together, our data suggest that large fluctuations in 5mC could be driving acute stress response, whereas 5mC loss could be necessary to reduce the “allostatic load” and achieve adaptive homeostasis. Furthermore, the degree of 5mC loss could be critical as stress persists in that resilient animals achieve 5mC levels closer to baseline. Regarding 5hmC, there were minimal alterations in its dynamics between acute, chronic, and longitudinal stress. This possibly indicates that once 5hmC is added or removed from a region, it functions as a permanent epigenetic/transcriptomic architectural rearrangement in response to stress.

An emerging component of stress habituation is the role epigenetic memory is playing, as it can impact gene expression acutely or chronically. Epigenetic memory, more specifically transcriptional memory, requires changes in the chromatin architecture that then allow the cells to amount a more robust response upon re-exposure (D’Urso and Brickner 2014). From our 5hmC ASDS analysis, we found that animals “primed” for stress susceptibility had reduced expression of chromatin remodeling complex subunits (*Arid1b, Arid2, Smarca2, Rest*, and *Ring1*). Animals confirmed to be susceptible after CSDS also exhibited a concomitant 5hmC reduction and repression of chromatin (*Smarca2*) and histone (*Smyd1, Rcor2,* and *Pwwp2a*) modifying genes. Furthermore, in our depression susceptible model where 5hmC continuously decreased along the ASDS-CSDS-LSDS continuum, the DNA modifying proteins Dnmt3a, Tet2, and Tet3 were found. Cumulatively, these data propose a mechanism whereby depression susceptibility could be connected to insufficient expression of the epigenetic machinery necessary to promote chromatin rearrangement and acquire the epigenetic memory that would allow for a resilient stress response. On the contrary, resilient animals appear to be “primed” through the activation or protection of adult neuro/oligo-genesis as independently observed in all three stress paradigms. Although chronic stress is known to impair adult neurogenesis (Jones *et al*. 2022), studies have shown that if this process can be protected, it can actually defend against stress susceptibility (Schloesser *et al*. 2010; Hill *et al*. 2015). Our findings that stress-resilient animals exhibit upregulation of genes that facilitate adult neurogenesis and oligodendrocyte precursor cells support these findings and suggest that these mechanisms could serve as a critical contributor toward stress resilience.

The monoaminergic neurotransmitter hypothesis for MDD has dominated the field of study and still continues to be the primary source of available treatment options (Niciu *et al*. 2014). This is likely attributed to the fact that serotonin, norepinephrine, and dopamine levels and their relationship with mood (focus/calmness, alternes/energy, anhedonia/pleasure, respectively) make them effective targets for treatment (Nutt 2008). Interestingly, our data showed a strong bias toward GABAergic and glutamatergic synapse function, composition, and release compared to other synapses, particularly in resilient animals. In particular, in the L_RR_ and CSDS resilient gained DMRs overlap, we observed an enrichment in synaptic vesicle cycling and ionotropic glutamate receptor signaling processes. During chronic stress, glutamate uptake and synaptic clearance are impaired by several factors: the loss of glutamate receptors on postsynapses and glial cells and/or impaired receptor cycling (Popoli *et al*. 2012). This results in a spillover effect whereby extrasynaptic glutamate receptors become activated and induce downstream cell death signaling (Hardingham and Bading 2010). Our data suggest that 5mC and 5hmC could be encouraging resilience by managing glutamatergic synapses and protecting against these neurotoxic effects. Importantly, both glutamate and GABA have been observed to contribute to MDD behaviors in prior experiments and are receiving more immediate attention as treatment options for MDD (Werner and Covenas 2010; Niciu *et al*. 2014).

In addition to GABA and glutamate, our results also uncovered a role for adenosine in modulating stress response. In the CNS, adenosine functions in a neuroprotective manner by preventing its own accumulation during ischemia, glutamate excitotoxicity, or seizures (Wardas 2002). Moreover, adenosine receptors are present in dopaminergic and glutamatergic neurons and co-localize with various neurotransmitter receptors. Intriguingly, we identified ASDS-specific DMRs in the neuronal calcium-binding protein, *Necab2*, the high-affinity adenosine receptor, *Adora2*, and the dopamine receptor subunit, *Drd2*. Both the dopaminergic and adenosinergic systems have been implicated in anxiety disorders (Fraporti *et al*. 2019). Moreover, Necab2 interacts with and controls the cell surface expression and function of Adora2a (Canela *et al*. 2007), which directly interacts with Drd2 forming heterodimers, dampening the binding affinity Drd2 has for dopamine (Ferre *et al*. 1991). This protein network insinuates a mechanism whereby Necab2—Adora2a interaction reduces Adora2a plasma membrane expression, creating an environment where more dopamine can bind to its receptor and potentially protect against anxiety-like behaviors. Further supporting the role of adenosine in stress-induced depression, we also identified three adenosine deaminases (*Ada*, *Adarb1*, and *Adarb2*) in our CSDS and ASDS-CSDS-LSDS analyses. In addition to their neuroprotective properties, *Adarb1/2* targets the pre-mRNA of the GABA_A_ receptor (Ohlson *et al*. 2007), and serotonin receptor (Burns *et al*. 1997; Di Narzo *et al*. 2014) for A-to-I editing, drastically affecting the affinity for neurotransmitter binding.

Our results indicate that stress-susceptible animals show patterns of genetic dysregulation associated with compromised neuro-vasculature system, impaired BBB function, altered lipid metabolism, and microglia-mediated neuroinflammation. These phenotypes are reminiscent of early AD pathology and support the hypothesis that exposure to chronic stress is a major risk factor for AD (Machado *et al*. 2014). In both concomitantly increasing and decreasing 5hmC susceptible genes identified from our CSDS analysis, we found many genes associated with later-stage AD pathology. For example, *Prkag2*, *Dctn6, Bag3*, and *Rer1* have been linked to A β -plaques (Kaether *et al*. 2007; Sturner and Behl 2017; Bharadwaj and Martins 2020; Sharoar *et al*. 2021), *Hs3st1* with neurodegeneration (Tan *et al*. 2021) and *Lgmn* and *Ppp2cb* with neurofibrillary tangles (Zhang *et al*. 2014; Jun *et al*. 2022). Furthermore, the 5hmC dynamics across the ASDS-CSDS-LSDS timeline revealed *G3bp2*, *Grhl1*, *Flt4*, and *Pecam1*, all of which been previously associated with AD [100–103]. These findings point to an increase in the severity of AD-related gene expression changes across our epigenetic stress timeline. Overall, our results suggest that changes to the DNA modification landscape could potentially be part of the mechanism through which chronic stress creates the characteristic cellular pathology associated with AD in stress-susceptible individuals.

An interesting question not addressed in our analysis is how resilience is lost over time? In addition to the SS and RR animals identified in the LSDS analysis, we also observed a third group, resilient animals that became susceptible after stress re-exposure (data not shown). Initial investigations into differential regions and corresponding biological pathways and genes affected by stress-induced alterations in 5mC and 5hmC were strikingly similar to RR and SS animals. This suggests that other mechanisms are likely at hand.

In conclusion, our study expands upon previous research and provides a much-needed global and comprehensive perspective on the contributions from DNA modifications in stress-induced depression. Our study is the first to simultaneously profile paired 5mC, 5hmC, and transcriptome data on the genome-wide scale across three different durations of social defeat. This allowed us to investigate the dynamics of DNA modifications in relation to stress and offer potential therapeutic targets for MDD.

## Supplementary Material

jkad114_Supplementary_Data

## Data Availability

All genome-wide sequencing datasets, including 5mC-seq, 5hmC-seq and RNA-seq raw and processed have been deposited to Gene Expression Omnibus (GEO) repository (GSE223301). We deposited our gene expression datasets for Supplementary Figures 3c, 3d, 4d, 4e, 5d, and 5e into Mendeley Data (https://data.mendeley.com/datasets/dpd6x6y969). Supplemental material available at G3 online.
