# Supplementary material for: Social defeat stress induces genome-wide 5mC and 5hmC alterations in the mouse brain
Source: G3 (Bethesda). 2023 May 25;13(8):jkad114. doi: 10.1093/g3journal/jkad114 (PMC10411578; doi:10.1093/g3journal/jkad114)
Supplement: jkad114_Supplementary_Data [file jkad114_supplementary_data.zip › Supplemental_Figure_Legends_G3-2023-404270.docx]

**Figure S1:** **Chronic social defeat stress induces social avoidance in young and mature adult mice**

**A** and **B.** 3- and 6-month animals daily nest building.

**C and D.** 3- and 6-month animals daily food consumption *(*P < 0.05, **P < 0.01 and ***P < 0.001, ****P < 0.0001 in Holm-Sidak method for multiple comparisons after unpaired t-test).*

**E** and **F.** Time spent in the interaction zone (**E**) and avoidant zone (**F**) when target was either absent (-) or present (+) *(*P < 0.05, **P < 0.01 and ***P < 0.001, ****P < 0.0001 in Tukey’s post hoc test after One-way ANOVA*).

*Abbreviations: Ctrl: control, Str: Stress, n.s.: not significant, Med: Medium*

**Figure S2: Characterization of susceptible and resilient chronic stress-induced DMRs and DhMRs**

**A-D.** Enrichment of 5mC (**A** and **B**) and 5hmC (**C** and **D**) peaks at 3’ and 5’ untranslated regions (3’ and 5’UTR), promoters, exons, introns, transcription termination sites (TTS) and intergenic regions.

**E and F.** Overlap of 5mC and 5hmC differential regions demonstrating the presence of 5hmC is dispensable of 5mC.

**G.** Box plot show percentage of concomitant ratio for 5mC accumulated region (gain DmR), 5mC depleted region (loss DmR), 5hmC accumulated region (gain DhmR) and 5hmC depleted region (loss DhmR).

**H.** Scatter plot show correlation of 5hmC change and gene expression change of 5hmC depleted regions for 3-month susceptible mouse.

**I.** Pie chart show number of DhmR that are concomitant with gene expression in 5hmC depleted regions of 3-month susceptible mouse.

*Abbreviations: Ctrl: Control, Sus: Susceptible, Res: Resilient, mC: 5mC, hmC: 5hmC*

**Figure S3: Comparison of shared DMRs and DhMRs in 3- and 6-month animals**

**A-H.** Normalized 5mC or 5hmC counts at specific peak regions in 6-month (**A-D**) CSDS animals: *Drd*2 (**A**), *Tph2* (**B**), *Gria2* (**C**) and *Kl* (**D**) and 3-month (**E-H.**) CSDS animals: *Per2* (**E**), *Pvalb* (**F**), *Foxo1* (**G**) and *Sesn3* (**H**).

**I** and **J.** 5mC (**I**) and 5hmC (**J**) regions that are gained or lost in both 3- and 6-month susceptible or resilient animals. 6-month CSDS animals gain and lose 5mC and 5hmC to the same magnitude as 3-month CSDS animals.

**Figure S4: Acute social defeat stress is sufficient to induce social avoidant-like behavior**

**A.** Experimental design of ASDS paradigm, behavior tests and tissue and blood collection.

**B.** Daily nest building.

**C.** Daily food consumption *(***P < 0.001 in Holm-Sidak method for multiple comparisons after unpaired t-test).*

**D.** ASDS causes a social avoidant-like phenotype. (Unpaired t-test, *****P < 0.0001*). Red dots indicate randomly selected animals used for downstream 5mC and 5hmC enrichment and RNA-seq experiments.

**E.** Sucrose preference (*Unpaired t-test, ***P < 0.001*).

**F** and **G.** Blood corticosterone was significantly elevated in stressed animals immediately following ASDS (**F**), but not 36 hours post stress (**G**) (*Unpaired t-test, **P < 0.01*).

**H-I.** Enrichment of 5mC (**H**) and 5hmC (**I**) peaks at 3’ and 5’ untranslated regions (3’ and 5’UTR), promoters, exons, introns, transcription termination sites (TTS) and intergenic regions.

**J-L.** Normalized 5mC counts at peak regions identified in ASDS animals: *Necab2* (**J**), *Adora2a* (**K**), *Drd2* (**L**).

*Abbreviations: Ctrl: Control, SD: Social Defeat, SI: Social Interaction, Str.: Stress, TSS: Transcription Start Site, TES: Transcription End Site*

**Figure S5: Longitudinal Social Defeat Stress**

**A.** Experimental design of LSDS paradigm and tissue and blood collection.

**B.** SI data subcategorizing animals into L_SS_ (Susceptible remained susceptible) or L_RR_ (Resilient remained resilient).

**C.** Number of 5mC and 5hmC peaks identified in control and longitudinally stressed animals.

**D** and **E.** Enrichment of 5mC and 5hmC peaks at 3’ and 5’ untranslated regions (3’ and 5’UTR), promoters, exons, introns, transcription termination sites (TTS) and intergenic regions.

**F.** Average normalized 5hmC read count across ASDS, CSDS and LSDS susceptible (top) or resilient (bottom) continuum with continual 5hmC accumulation (Group I), depletion (Group II), or a return to base line (Groups III and IV).

**G.** Number of genes harboring both 5mC/5hmC susceptible/resilient regions from Groups I-IV.

*Abbreviations: SI: social interaction, SS: Susceptible remained susceptible, RS: Resilient to susceptible, RR: Resilient remained resilient, TSS: Transcription Start Site, TES: Transcription Termination Site*
