# Supplementary figures and images for: Social defeat stress induces genome-wide 5mC and 5hmC alterations in the mouse brain
Source: G3 (Bethesda). 2023 May 25;13(8):jkad114. doi: 10.1093/g3journal/jkad114 (PMC10411578; doi:10.1093/g3journal/jkad114)

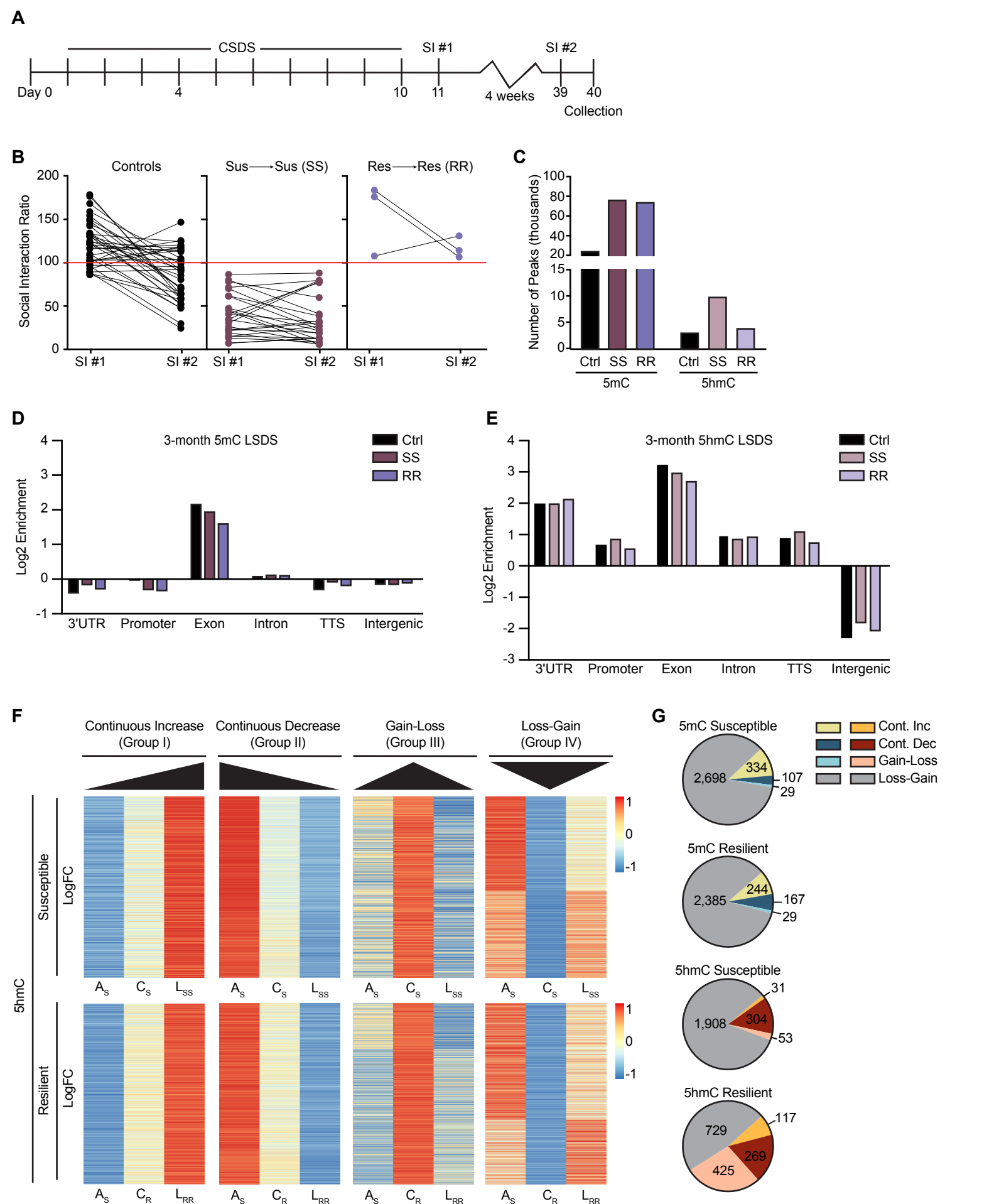

Sup. Figure 5

Supplement: jkad114_Supplementary_Data [file jkad114_supplementary_data.zip › Figure S5.pdf]

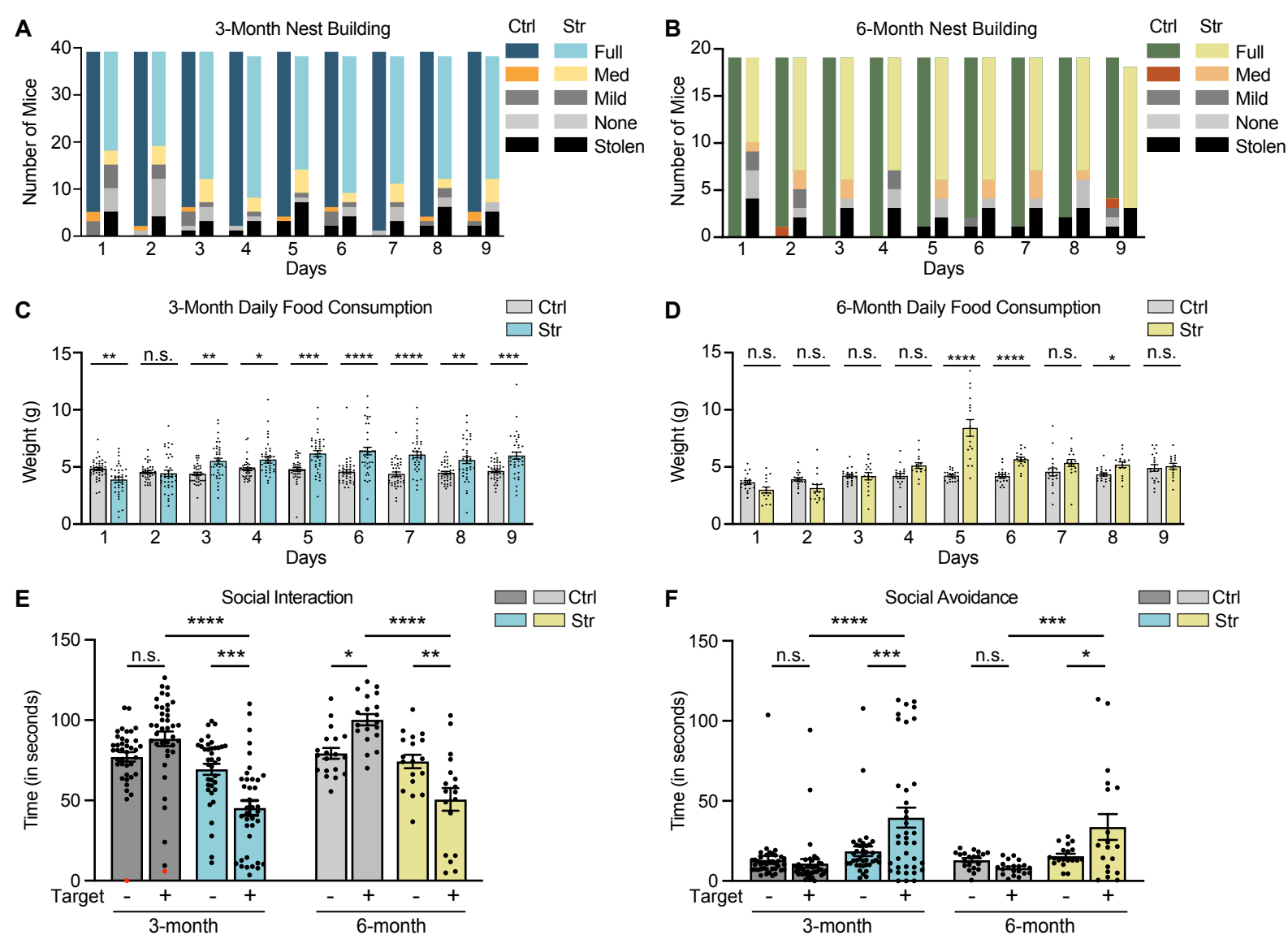

Supplement: jkad114_Supplementary_Data [file jkad114_supplementary_data.zip › Figure_S1_G3-2023-404270.pdf]

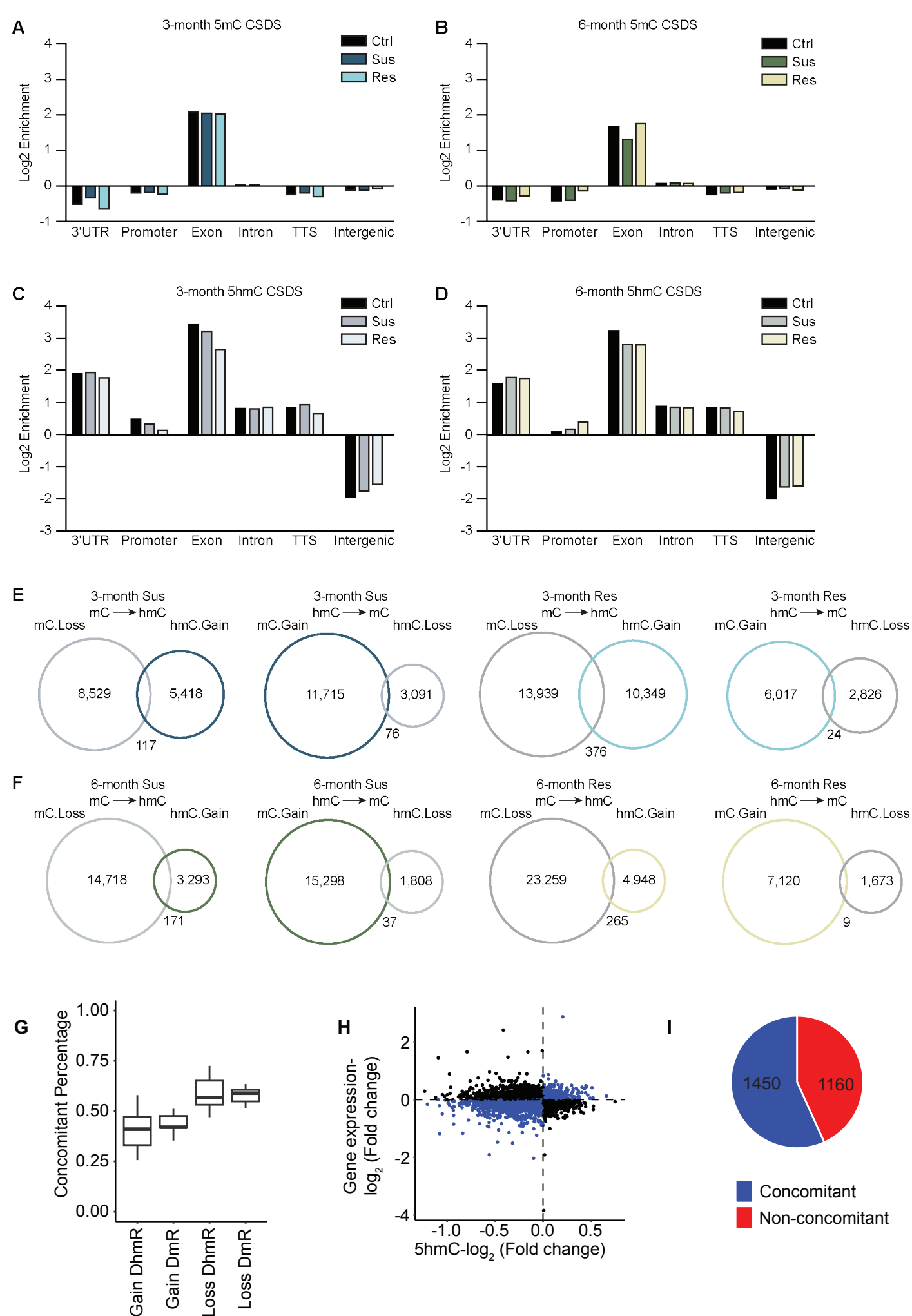

Sup. Figure 2

Supplement: jkad114_Supplementary_Data [file jkad114_supplementary_data.zip › Figure_S2_G3-2023-404270.pdf]

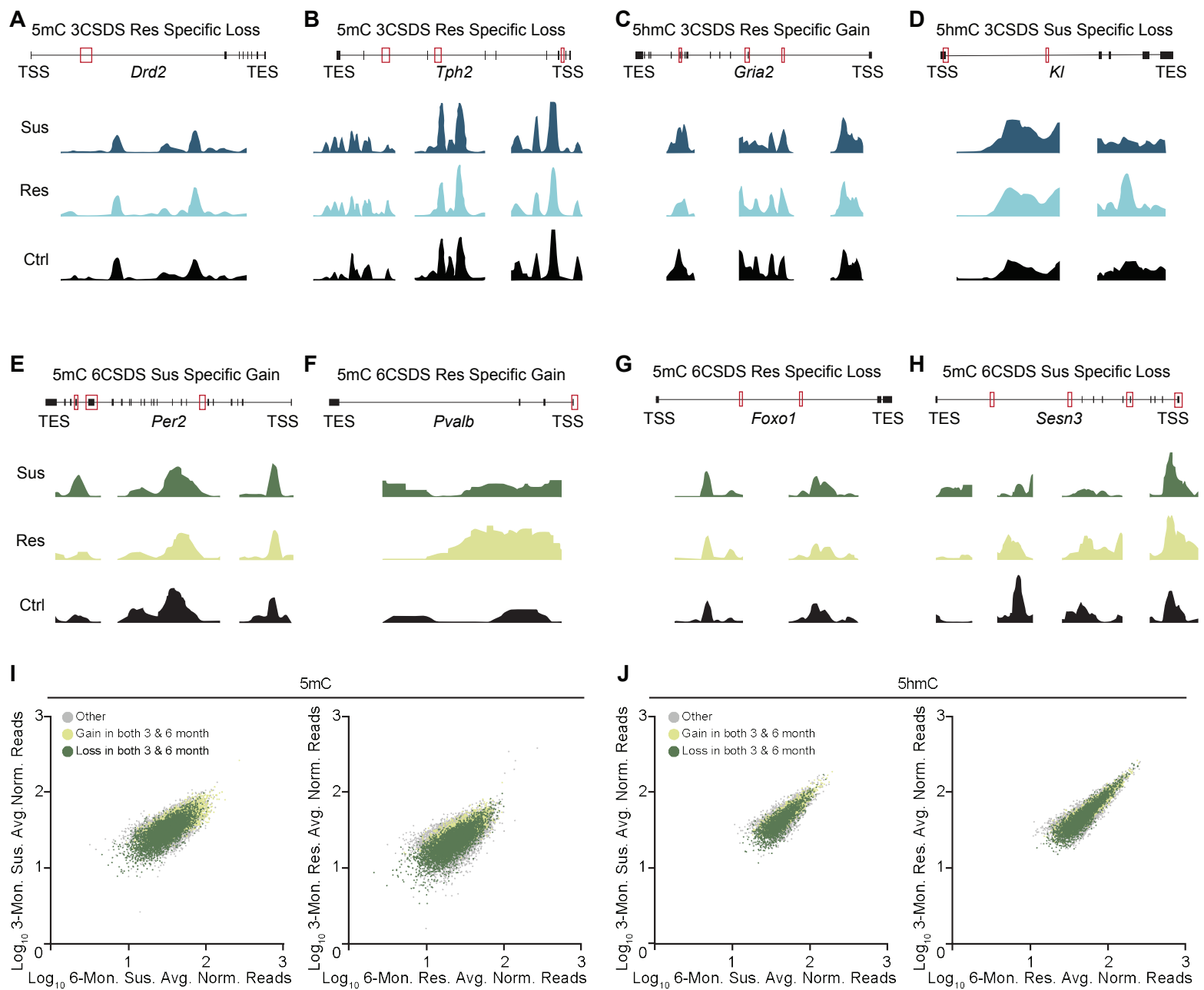

Supplement: jkad114_Supplementary_Data [file jkad114_supplementary_data.zip › Figure_S3_G3-2023-404270.pdf]

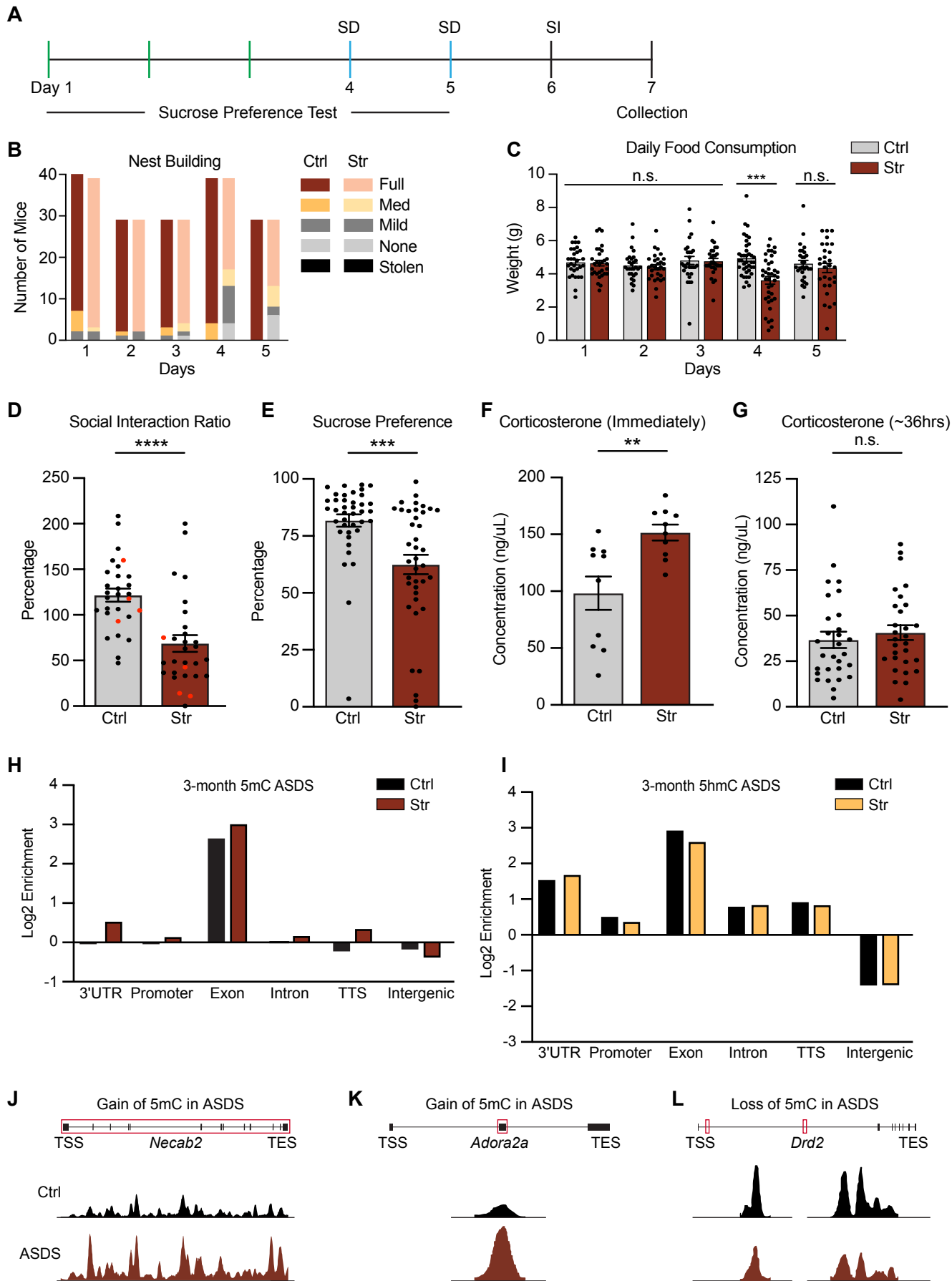

Supplement: jkad114_Supplementary_Data [file jkad114_supplementary_data.zip › Figure_S4_G3-2023-404270.pdf]
